# Evidence for Trace Gas Metabolism and Widespread Antibiotic Synthesis in an Abiotically Driven, Antarctic Soil Ecosystem

**DOI:** 10.1111/1758-2229.70249

**Published:** 2025-12-09

**Authors:** A. R. Thompson, B. J. Adams, I. D. Hogg, S. Yooseph

**Affiliations:** ^1^ Kravis Department of Integrated Sciences Claremont McKenna College Claremont California USA; ^2^ Ronin Institute Riverside California USA; ^3^ Department of Biology Brigham Young University Provo Utah USA; ^4^ Monte L. Bean Life Science Museum Provo Utah USA; ^5^ Canadian High Arctic Research Station, Polar Knowledge Canada Cambridge Bay Nunavut Canada; ^6^ School of Science University of Waikato Hamilton New Zealand

**Keywords:** Antarctic microbiology, bacterial community structure, carbon fixation pathways, McMurdo Dry Valleys (MDVs), metagenome‐assembled genomes (MAGs), microbial ecology, shotgun metagenomics, trace gas metabolism

## Abstract

The McMurdo Dry Valleys (MDVs) of Antarctica are a uniquely pristine, low‐biodiversity model system for understanding fundamental ecological phenomena, the impact of a warming climate on ecosystem functioning, community structure and composition and the dynamics of adaptation. Despite the scientific value of this system, we still know little about the functional ecology of its biota, especially the bacteria. Here, we analysed the bacterial taxonomic and functional diversity of 18 shotgun metagenomes using the VEBA metagenome processing pipeline. We recovered 701 medium‐to‐high quality metagenome‐assembled genomes (MAGs) (≥ 50% completeness and contamination < 10%) and 201 high‐quality MAGs (≥ 80% completeness and < 10% contamination), almost 50% more than found in similar sites previously. We found that: (1) community composition shifts along environmental gradients correlated with soil moisture, elevation and distance to the coast; (2) many MDV bacteria are capable of performing trace gas metabolism; (3) genes associated with antibiotic‐mediated competitive interactions (e.g., antibiotic biosynthesis and antibiotic resistance genes) are widespread; and (4) MDV bacteria employ survival strategies common to bacteria in similarly extreme environments. This study provides novel insight into microbial survival strategies in extreme environments and lays the groundwork for a more comprehensive understanding of the autecology of MDV bacteria.

## Graphical abstract

We analysed bacterial diversity in 18 shotgun metagenomes from a model soil ecosystem in Antarctica using the VEBA metagenome pipeline. We recovered 701 medium‐to‐high quality MAGs and found that these bacteria metabolise trace gases, produce potentially novel antibiotics and employ extremotolerance phenotypes to survive the extreme Antarctic soil environment.

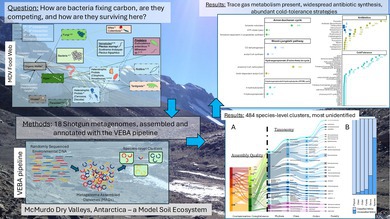

## 1 Introduction

The McMurdo Dry Valleys (MDVs) of Antarctica offer microbial ecologists an ideal model system for understanding fundamental ecological phenomena (Xue et al. 2024) and the effects of climate change on a microbial soil ecosystem (Fountain et al. 2014; Gooseff et al. 2017; Andriuzzi et al. 2018). The system is useful because of its reduced biotic diversity, its sensitivity to climate change and the decades‐long research focus by numerous research teams, including the United States National Science Foundation's McMurdo Dry Valley Long‐Term Ecological Research programme (MCM LTER). Because of the harsh climate, there are no vascular plants, only three species of arthropods (Adams et al. 2006), and most soils (> 95%) host only a single metazoan (the nematode *Scottnema lindsayae*) or none at all (Virginia and Wall 1999; Courtright et al. 2001; Bamforth et al. 2005). This is a system dominated by microbiota (Lee et al. 2012; Thompson et al. 2020). Soils are exceptionally arid (< 5% soil moisture) (Burkins et al. 2001), saline and oligotrophic, and as a polar desert the region is sensitive to climate change and likely to experience dramatic ecological changes sooner than many other ecosystems (Fountain et al. 2014; Gooseff et al. 2017). Moreover, research in these valleys has been ongoing for three decades and continues today with long‐term experiments and data archives spanning more than 25 years (Gooseff et al. 2017; Andriuzzi et al. 2018). Researchers have investigated the diversity, distribution, taxonomy and function of metazoans (Smith et al. 2012; Collins and Hogg 2016; Shaw et al. 2018), bacteria (Cary et al. 2010; Sokol et al. 2013; Buelow et al. 2016), fungi (Fell et al. 2006; Gokul et al. 2013), protists (Bamforth et al. 2005; Thompson et al. 2020), archaea (Magalhães et al. 2014; Richter et al. 2014) and viruses (Zablocki et al. 2014; Wei et al. 2015; Adriaenssens et al. 2017; Bezuidt et al. 2020) as well as regional and local scale edaphic, climatic, geological and hydrological parameters (Doran et al. 2002, 2010; Barrett et al. 2006; Simmons et al. 2009; Gooseff et al. 2017; Andriuzzi et al. 2018). Thus, MDV soils are a prime candidate for use as a comprehensive model of a soil ecosystem. However, key knowledge gaps remain regarding the functioning of these communities, limiting the full potential of this system as a model (Adams et al. 2006; Thompson et al. 2020, 2021; Xue et al. 2024). Perhaps most significantly, the genomic diversity and functional capabilities of its bacterial communities remain poorly understood.

Bacteria perform critical services to an ecosystem via nutrient cycling and community structuring processes (Sikorski 2015). These services are mediated by the diversity of a community's ecological functioning and life history strategies, many of which are influenced by bacterial phylogenetic and taxonomic diversity. Thus, characterising bacterial taxonomic and functional diversity can inform aspects of ecosystem functioning. It is well established that MDV soil communities exist along a climatic gradient that somewhat correlates with the distance from the coast and elevation and exerts some control over their composition and structure (Marchant and Head 2007; Dragone et al. 2022; Mashamaite et al. 2023). What is less clear is how this gradient impacts low‐level taxonomic diversity (genus and species) and functional attributes (Van Horn et al. 2013; Dragone et al. 2022), such as nutrient cycling and survival strategies. Understanding this relationship is important as previous research has predicted that as the MDVs warm due to climate change, the more arid soil communities will be replaced by taxa common in current moist soils (Andriuzzi et al. 2018), which may threaten the potentially unique taxonomic and functional diversity of arid MDV communities.

How the majority of MDV soil communities outside algal mats, moss beds and lithic environments is sourcing their carbon (whether from atmospheric trace gases, windblown mat material or legacy sources) is debated (Ortiz et al. 2021). Recent work has shown that trace gas metabolism—the incorporation of CO_2_, CO and H_2_ via non‐photosynthetic means—is widespread in soils globally (Bay et al. 2021) as well as in Antarctic sites similar to those of the MDV (Ji et al. 2017; Ortiz et al. 2021). However, to our knowledge, no study has confirmed this for sites that have been the focus of comprehensive characterisation. Nitrogen is also limited in the MDVs (Monteiro et al. 2020), with previous studies unable to recover nitrogen‐fixing microbes outside wetted margins of ponds and streams (Cary et al. 2010).

It is unclear how much MDV soil communities are structured by biotic and abiotic variables (Caruso et al. 2019; Lee et al. 2019; Thompson et al. 2021). Multi‐tiered trophic levels exist in MDV soils (Bamforth et al. 2005; Shaw et al. 2018; Thompson et al. 2021) and studies have identified antibiotic‐associated genes from various surveys (Fierer et al. 2012; Wei et al. 2015; Núñez‐Montero and Barrientos 2018). Whether bacteria are competing directly (via antagonistic interactions) or indirectly (via extremotolerance survival strategies) is still an unanswered question.

To corroborate prior estimates of MDV bacteria diversity and assess the role of MDV soil bacteria in ecosystem functioning across a climate gradient, we analysed a dataset of 18 soil metagenomes previously used to characterise the taxonomic diversity and functional roles of soil eukaryotes in the MDVs (Thompson et al. 2020, 2021). Here, we: (1) evaluate bacterial community structure and function across climatic gradients; (2) examine the genetic potential for bacterial trace gas metabolism and nitrogen and sulphur metabolism; (3) estimate the potential for antibiotic‐mediated antagonistic interactions; and (4) identify tolerance strategies that enable survival in the harsh Antarctic environment.

## 2 Methods

### 2.1 Soil Sample Collection, DNA Extraction and Sequencing

**FIGURE 1:**
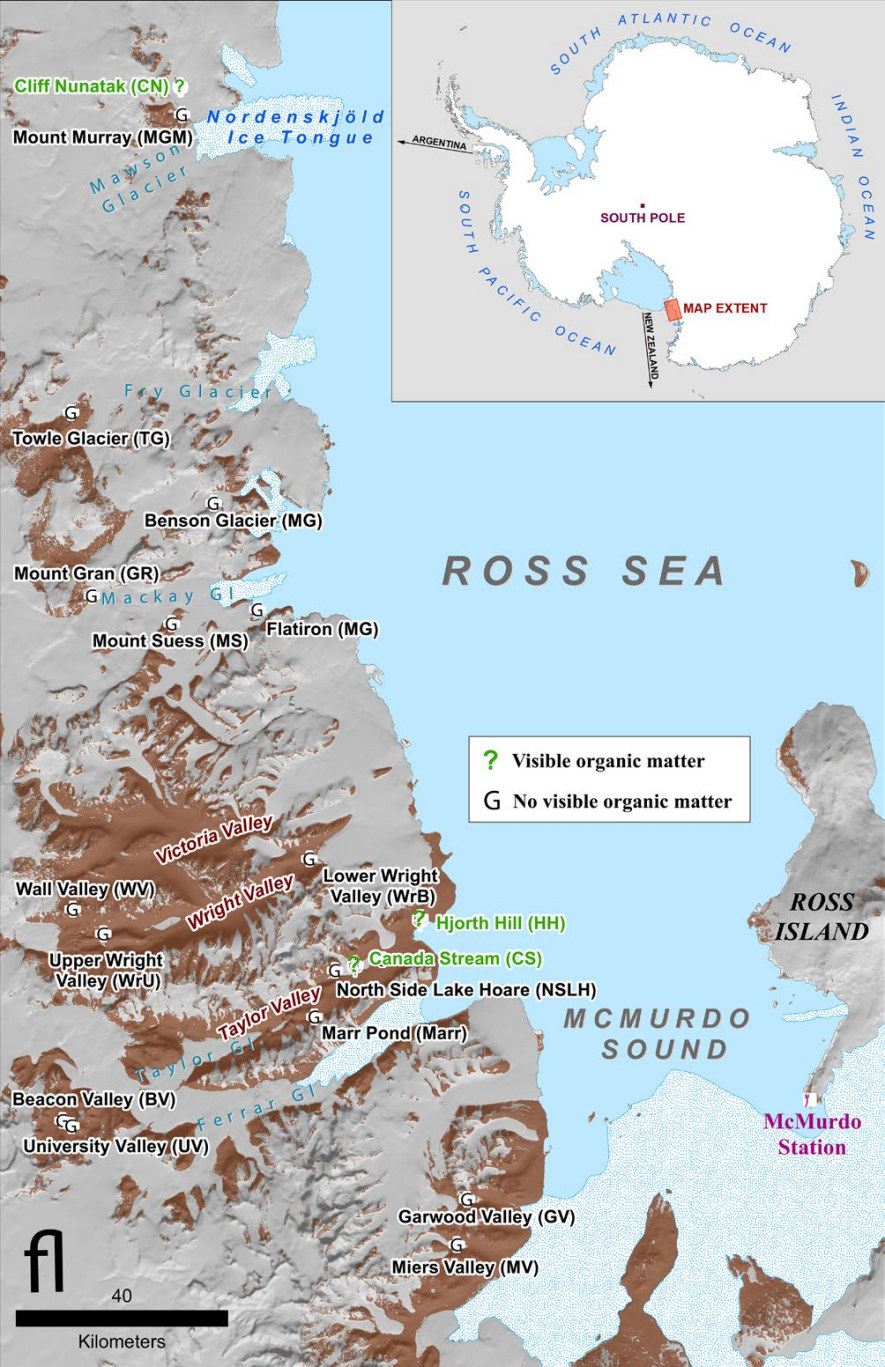
Map of study area (reproduced with permission from Thompson et al. 2020). The McMurdo Dry Valleys are located at roughly 77° S–162° E in Southern Victoria Land and open towards the Ross Sea to the East. Samples represent a variety of MDV soil habitats and landscape features that exist in the MDVs. Samples in green (Φ) possessed visible organic matter (e.g., moss and algae); samples in black were coarse to fine mineral soils with varying degrees of visible moisture. Figure by Mike Cloutier, Polar Geospatial Center.

Metagenomes were generated and analysed previously for their eukaryotic component (Thompson et al. 2020, 2021). Here we investigate the prokaryotic component of the metagenomes using genome mapping and the VEBA software for genome annotation (Espinoza and Dupont 2022; Espinoza et al. 2024). Full details on sampling and sequencing are provided in Thompson et al. (2020, 2021) and (Beet et al. 2016). Briefly, 87 soil samples were taken from 18 sites comprising a representative range of landscape types and features, latitudes and environmental gradients (Figure 1, Table S1). Most samples consisted of undeveloped mineral soils though several possessed varying degrees of vegetation (i.e., Hjorth Hill, moss; Cliff Nunatak, algae; and Canada Stream, biocrust). Samples were collected between 2014 and 2017 using sterilised scoops and sterile Whirl‐Pak bags and stored for transport and long‐term storage at −20°C. Subsamples were used to evaluate moisture content, pH, electrical conductivity, total N, total C, total P and NO_3_‐N (Figure S1). Texture and climatic zone were assigned to each site using available literature (Fountain et al. 2014; Thompson et al. 2020). Due to financial constraints, samples were pooled prior to DNA extraction, which was performed using the DNeasy PowerSoil Kit (Qiagen) with a protocol optimised for low biomass soils (see Thompson et al. 2020 for details). Libraries were prepared with the NEBNext Ultra II DNA Prep Kit (New England Biolabs) and sequenced on an Illumina HiSeq 2500 at a read length of 2 × 250 bp and a total insert length of 500 bp (Thompson et al. 2020). See Supporting Information for run stats (Table S2).

### 2.2 Genome Database Construction and Mapping

To assess the relative abundances of microbial genomes in our metagenomes, we mapped quality trimmed sequence reads from each sample to a collection of reference genomes (Jones et al. 2015; Loftus et al. 2021). Reads were trimmed using Trimmomatic with the settings LEADING 2, TRAILING 2, SLIDINGWINDOW 4:15 (default) and MINLEN 30 (Bolger et al. 2014; Thompson et al. 2020). For this analysis, we built a custom database using genomes sourced from publicly available databases, then mapped our reads to these genomes using Bowtie2 (Langmead and Salzberg 2012). The read mapping information was analysed using a probabilistic framework based on a mixture model to estimate the relative copy number of each reference genome in a sample (Xia et al. 2011; Loftus et al. 2021). To build the reference database, we used refseq (O'Leary et al. 2016), JGI GOLD (Mukherjee et al. 2025) and a Google Scholar literature search for Antarctic prokaryote genomes using combinations of the following characteristics: ‘soil’, ‘desert’, ‘cold desert’, ‘antarctic’, ‘polar’, ‘psychrotolerant’ and ‘assemble’. We included genomes from maritime Antarctic islands and Antarctic marine samples because there is overlapping diversity between continental, maritime and marine zones (Cary et al. 2010; Varliero et al. 2024). We used NCBI's batch download, filtered for complete genomes and those with annotations, excluded anomalous assemblies, those from metagenomes, and from unverified source organisms. Our resulting database (after removing duplicate genomes) contained 79,686 total genomes (78,082 refseq Bacteria genomes, 1066 refseq Archaea genomes, 526 genomes from the JGI Environment search and 12 from the Antarctic literature search).

### 2.3 Metagenome Processing

Raw, demultiplexed sequences were processed using the Viral Eukaryotic Bacterial Archaea (VEBA) open‐source pipeline, an end‐to‐end metagenomics software that can preprocess, assemble, cluster, classify and produce preliminary statistics on metagenomes from all domains of life and viruses (Espinoza and Dupont 2022; Espinoza et al. 2024). The pipeline was run with default settings for all modules. Briefly, sequences were trimmed using *fastp* (Chen et al. 2018) and assembled using *metaSPAdes* (Nurk et al. 2017). Unassembled reads were next mapped back to the assemblies using *Bowtie2* (Langmead and Salzberg 2012), and then *SeqKit* (Shen et al. 2016) was used to assess assembly statistics. Binning was performed by integrating multiple group‐specific software to optimise functionality and accuracy. For prokaryotes, *CoverM* (Aroney et al. 2024) was used to estimate coverage; *MaxBin2*, *MetaBat2* and *CONCOCT* were used for binning (Alneberg et al. 2014; Wu et al. 2016; Aramaki et al. 2019); *DAS Tool* was used for dereplication (Sieber et al. 2018); *Tiara* was used for filtering out non‐prokaryotic sequences (Karlicki et al. 2022); *CheckM* was used for quality assessment and filtering (Parks et al. 2015); and *Prodigal* and GTDB‐Tk were used for gene calling and genome classification, respectively (Hyatt et al. 2010; Chaumeil et al. 2020). VEBA removed poor quality MAGs according to its defaults: completeness ≤ 50% or contamination > 10 (Espinoza and Dupont 2022). MAGs were subsequently clustered into species‐level clusters (SLCs) at a 95% threshold (default) using *FastANI* (Jain et al. 2018). To annotate MAG proteins, *Diamond* (Buchfink et al. 2015) was used to align protein sequences against the NCBI non‐redundant protein database (default) while protein domains were identified using HMMER (Mistry et al. 2013) and the *Pfam* database (Mistry et al. 2021) and KEGG orthology was assessed using *KOFAMSCAN* (Aramaki et al. 2020). Finally, reads were mapped back to assemblies to estimate gene feature counts using the index building and mapping features in *Bowtie2*. The pipeline also processed contigs identified as eukaryotic or viral, though these data were not analysed for this study.

### 2.4 Analyses

**FIGURE 2:**
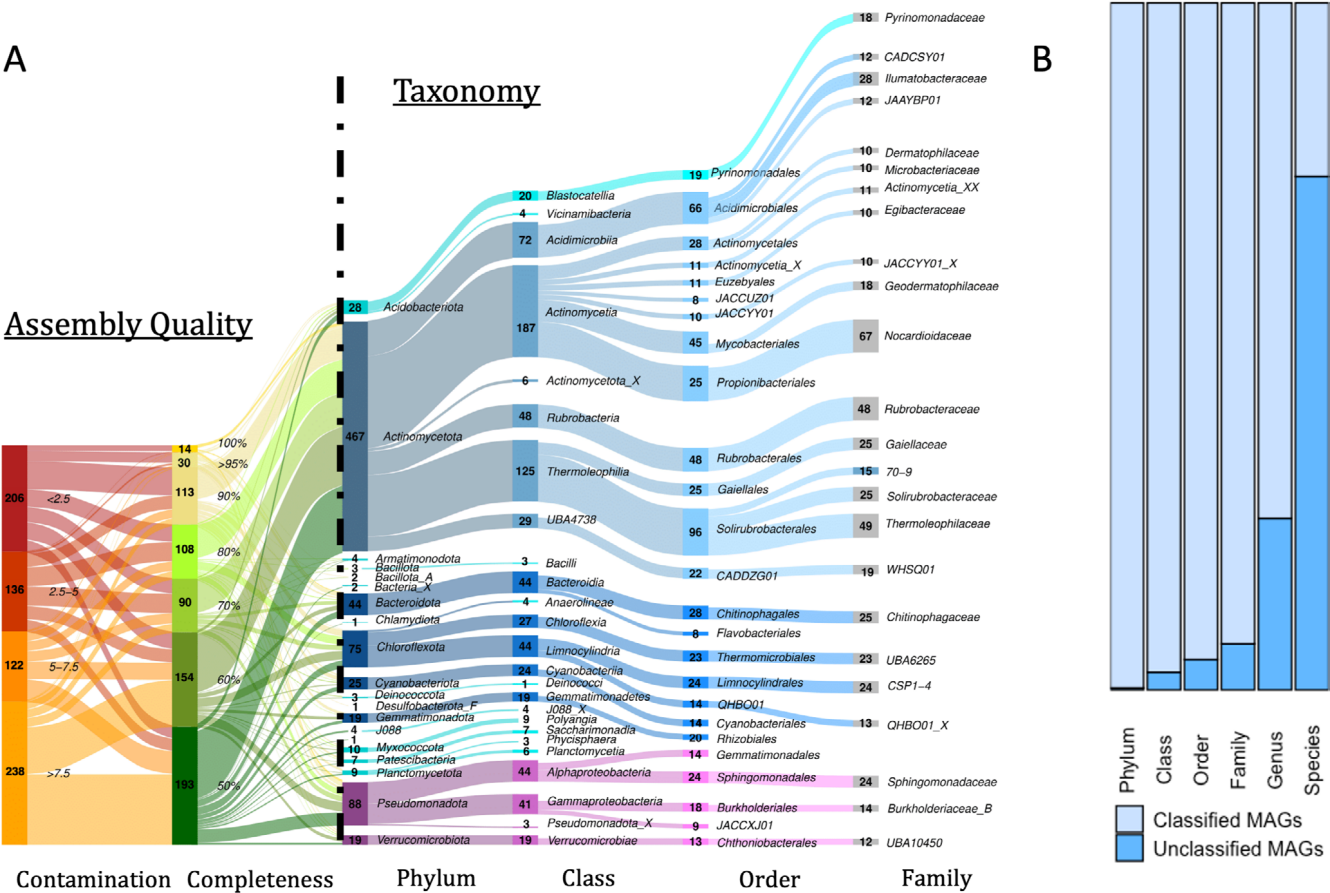
MAG taxonomy and assembly stats. (A) Sankey diagram of MAG taxonomic diversity coupled to assembly completeness (yellows and greens) and contamination level (reds and oranges). Only the top 20 taxa are shown for each level, and genera and species were excluded from the visualisation for clarity. Completeness percentages and contamination levels are shown in italics to the right of each category's counts, respectively. Completeness percentages represent ranges; for example, ‘50%’ includes all assemblies from 50% (inclusive) to 60%. (exclusive). (B) Proportion of classified to unclassified MAGs for each taxonomic level. Unclassified MAGs are defined as lacking a classification in the VEBA output and are displayed as ‘taxon_X’ in the Sankey diagram.

All analyses were conducted in R version 4.4.1 (R Core Team 2024) and plots were generated using ggplot2 version 3.5.1 (Wickham 2016) unless specified otherwise. To visualise MAG taxonomic diversity and assembly quality, we created a Sankey diagram (Figure 2) with the ggalluvial R package, version 0.12.5 (Brunson and Read 2023). Assembly quality stats and taxonomic classifications for the Sankey diagram were sourced from the CheckM and taxonomic_classification files produced by VEBA (Espinoza et al. 2024), which were combined then filtered for the top 25 taxa at each taxonomic level.

**FIGURE 3:**
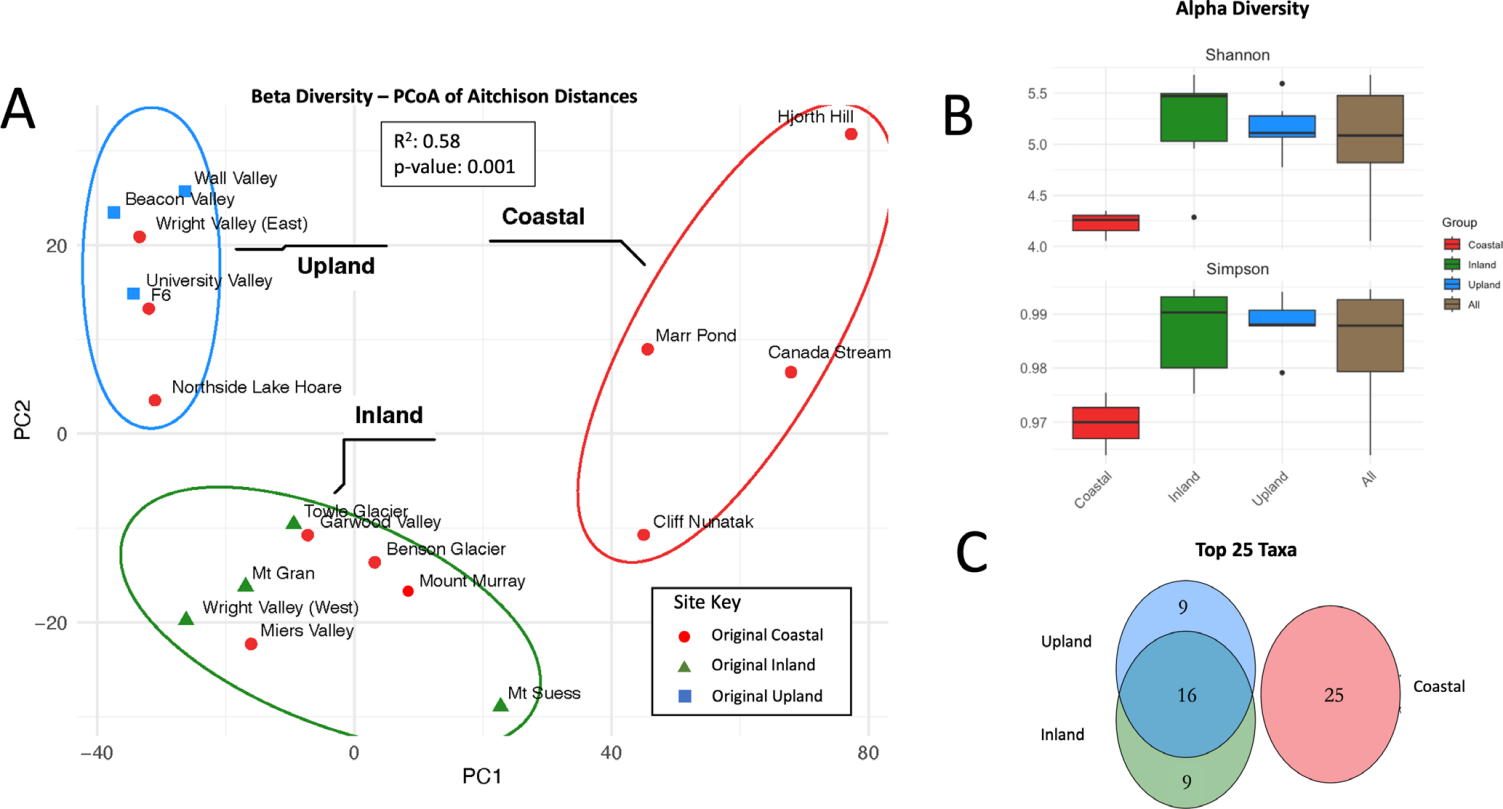
Diversity metrics and taxonomic overlap. (A) Beta diversity: PCA of site dissimilarity based on clr‐transformed MAGs (species‐level; SLCs), coloured by climatic zones: Coastal—red; inland—green, upland—blue. Site icons designate previous climactic designations: Coastal—red circle; inland—green triangle; upland—blue square. Ellipses around sites represent statistically significant site groupings. (B) Alpha diversity by zone and total (Shannon and Simpson's indices) using relativised counts. (C) Venn diagram showing overlap between top 25 most abundant taxa by climatic zone.

**FIGURE 4:**
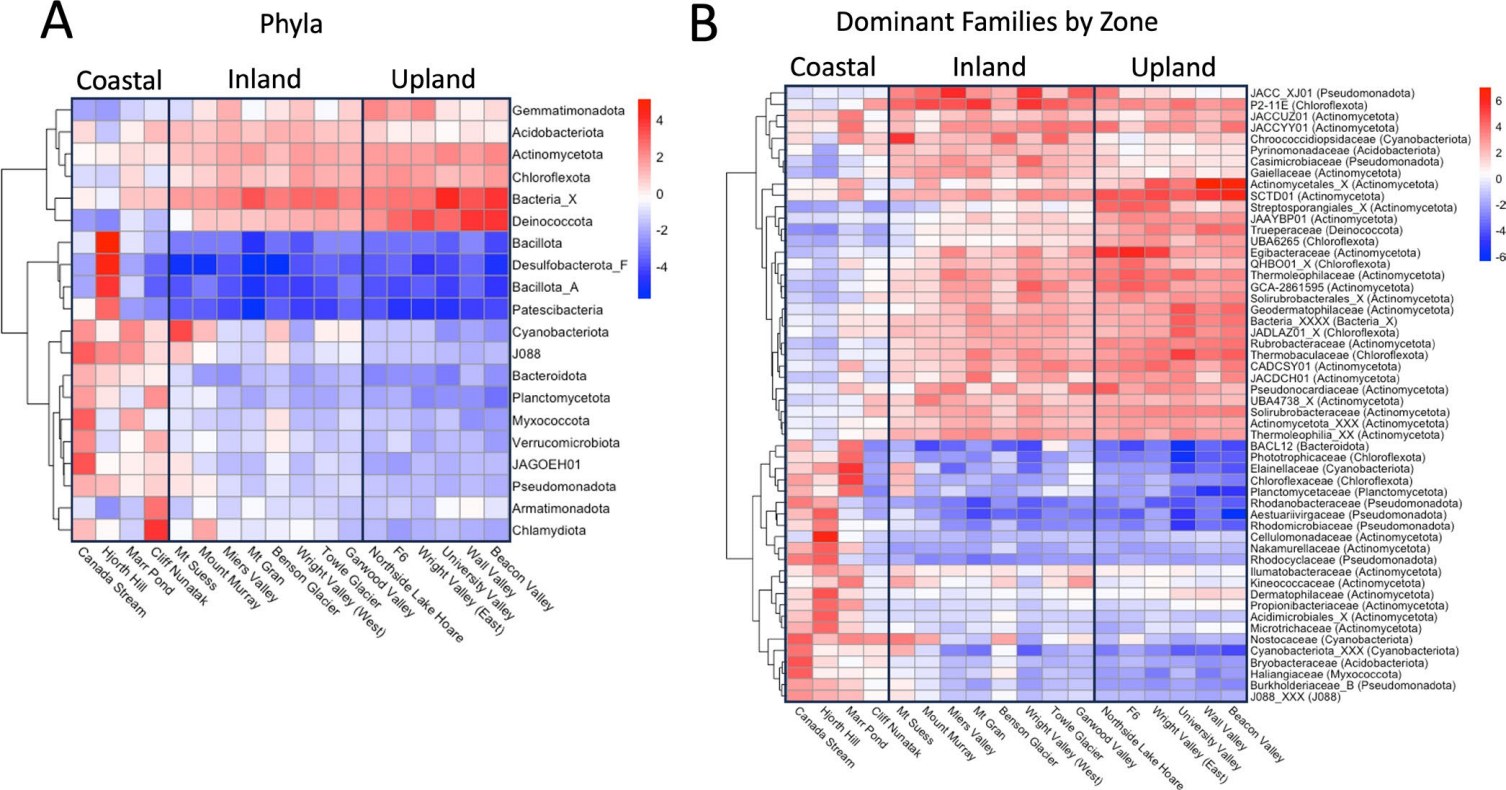
Heatmap of clustered MAGs (species‐level, SLCs) counts against sites at phylum (A) and family levels (B). Family levels represent top 50 most relatively abundant taxa in each climatic zone and taxon labels are family names followed by phylum in parentheses. Sites are grouped by climate groupings as estimated in Figure 3 (left to right: Coastal, inland and upland). Made using CLR‐transformed MAG counts data.

To visualise community dissimilarity, PCAs of CLR‐transformed species‐level clustered MAG and ORF counts were made using the prcomp() function in base R (Figure 3). To visualise the relative abundance of taxa, heatmaps of CLR‐transformed (Nearing et al. 2022) species‐level clustered MAG counts were made using pheatmap version 1.0.12 (Kolde 2018) (Figure 4).

**FIGURE 5:**
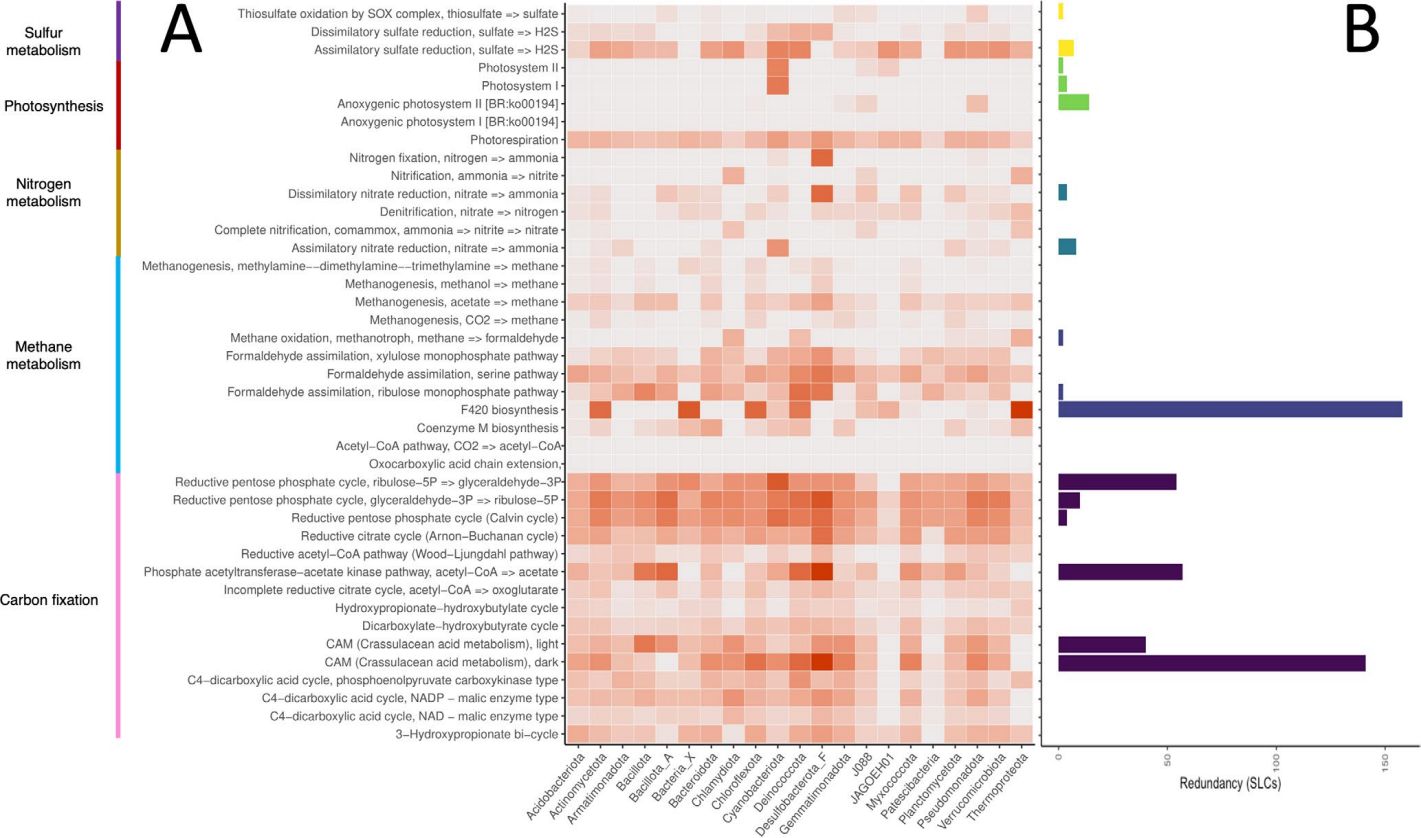
Completion and redundancy of KEGG modules associated with metabolism of key nutrients. (A) Mean completion of each pathway module by bacterial phylum. The darker the orange, the closer completion ratio is to 100%. (B) Number of SLCs with 100% complete KEGG gene pathways by module, a proxy for functional redundancy. Blank rows indicate no SLC possessed that pathway to 100% completion in any site. *x*‐axis represents phyla and taxonomic count, respectively. *y*‐axis represents KEGG pathways and modules.

**FIGURE 6:**
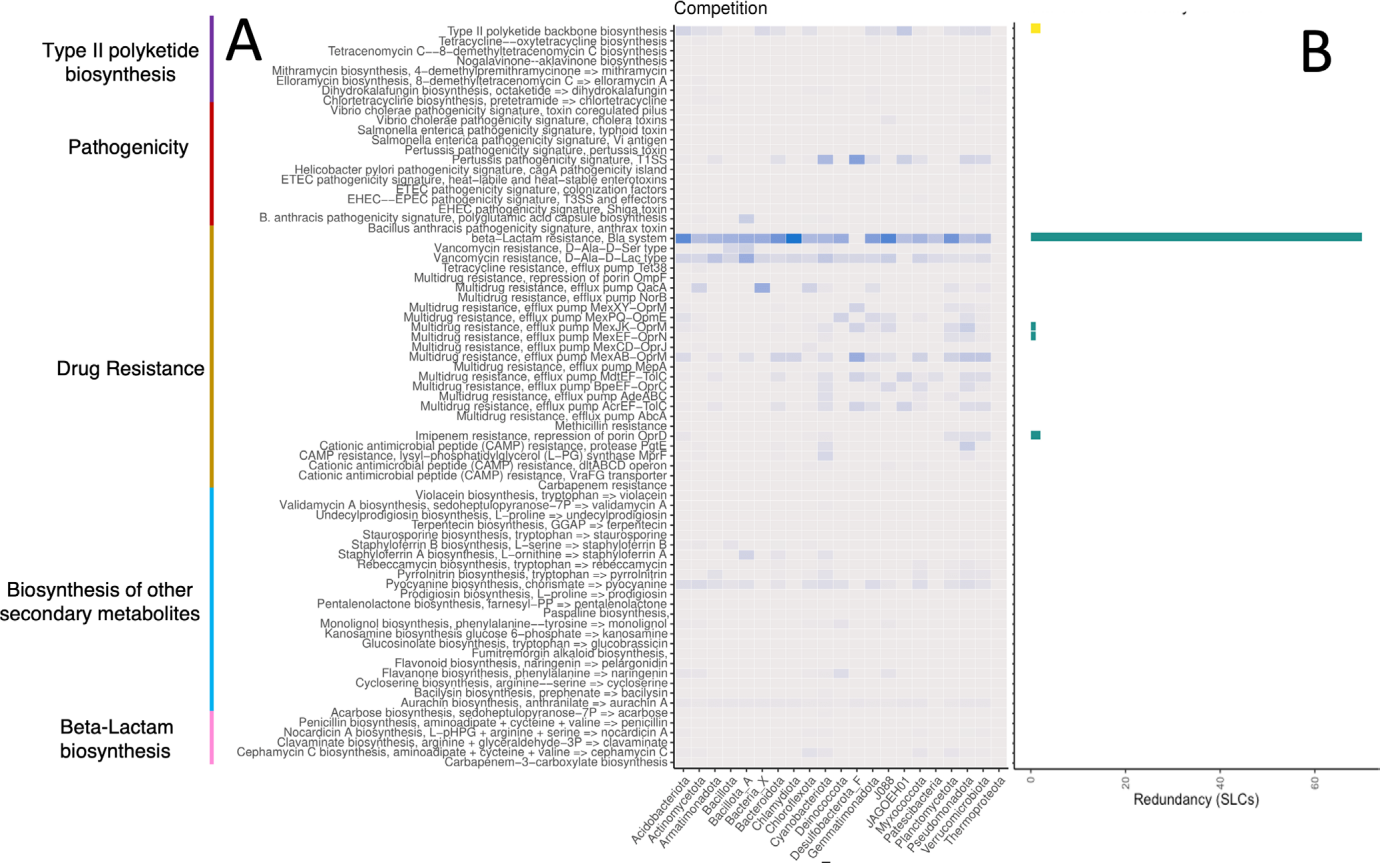
Completion and redundancy of KEGG modules associated with interspecies competition. (A) Mean completion of each pathway module by bacterial phylum. The darker the blue, the closer completion ratio is to 100%. (B) Number of SLCs with 100% complete KEGG gene pathways by module, a proxy for functional redundancy. Blank rows indicate no SLC possessed that pathway to 100% completion in any site. *x*‐axis represents phyla and taxonomic count, respectively. *y*‐axis represents KEGG pathways and modules.

**FIGURE 7:**
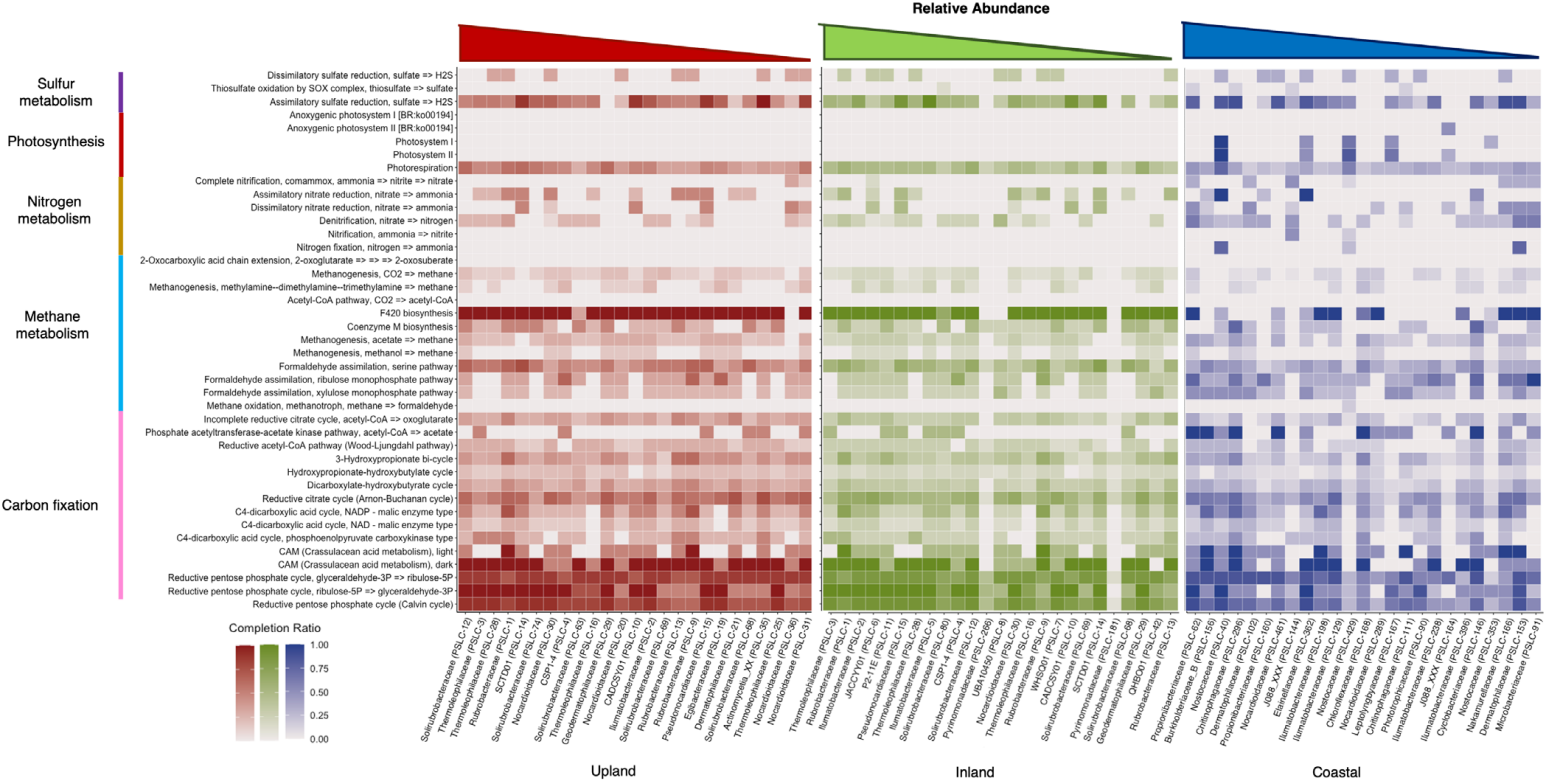
Metabolic functional differences between climatic zones (for top 25 abundant taxa). Functional differences are estimated using completion ratios of KEGG modules displayed as heatmaps. Climatic zones are as follows: Inland (red), upland (green) and coastal (blue). Taxa (*x*‐axis) represent the most relatively abundant taxa by zone (based on clr‐transformed SLC counts) with relative abundance increasing towards the left side of each graph. Taxa are listed with family followed by the unique SLC taxon id assigned by VEBA (e.g., PSLC‐1) for convenience in understanding their functional ecology and for comparing dominant taxa composition across zones. *y*‐axis shows KEGG pathways (far left) and select module names for each pathway (near left).

**FIGURE 8:**
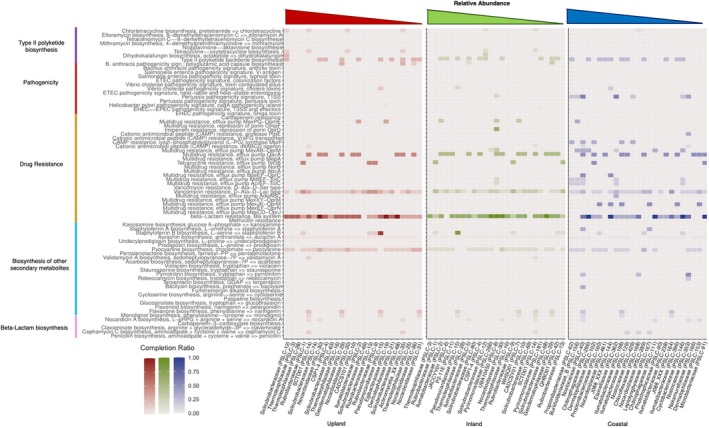
Competitive functional differences between climatic zones (for top 25 abundant taxa). Functional differences are estimated using completion ratios of KEGG modules displayed as heatmaps. Climatic zones are as follows: Inland (red), upland (green) and coastal (blue). Taxa (*x*‐axis) represent the most relatively abundant taxa by zone (based on clr‐transformed SLC counts) with relative abundance increasing towards the left side of each graph. Taxa are listed with family followed by the unique SLC taxon ID assigned by VEBA (e.g., PSLC‐1) for convenience in understanding their functional ecology and for comparing dominant taxa composition across zones. *y*‐axis shows KEGG pathways (far left) and select module names for each pathway (near left).

KEGG pathway completion ratios produced by VEBA were used to produce Figures 5, 6, 7, 8. Mean completion values for each pathway were calculated by averaging completion ratios for each listed pathway across sites (either all sites for Figures 5 and 6 or sites in each climate zone for Figures 7 and 8). For climate zone specific Figures 7 and 8, the top 25 most abundant taxa from each zone were used (Figure S2). KEGG pathways were restricted to those involved in potentially ecologically significant behaviours including virulence factors, extremotolerance, competition or nutrient cycling, using the literature.

A literature search was performed to identify keywords of genes and phenotypes involved in desiccation tolerance, cold shock response and tolerance, osmotic stress tolerance, radiation tolerance, antibiotic resistance, antibiotic synthesis, virulence factors (Chandra and Kumar 2017; Cycoń et al. 2019) and the six known pathways for fixing carbon (e.g., Calvin–Benson cycle and Arnon–Buchanan cycle; see Berg 2011). The list of keywords resulting from the search (Table S3) was queried against our gene annotation tables to estimate phenotype frequency in our MAGs.

## 3 Results

### 3.1 Taxonomic Diversity

Using default settings, VEBA recovered 701 medium‐to‐high quality bacterial MAGs (completeness > 50% and contamination < 10%) and 201 high quality bacterial MAGs (completeness > 80% and contamination < 10%). The 701 medium‐to‐high quality MAGs clustered to 485 species‐level MAG clusters (SLCs) in 20 phyla, 38 classes, 82 orders and 140 families. Most MAGs (75%) were unidentifiable at the species level. However, 75% corresponded to previously identified genera and 93% to previously identified families. There was a negative relationship between genome contamination and completeness (−0.109, *R* ^2^ = 0.2779, *p*‐value = < 2e‐16; Figure S3) and the average genome size was 3336 genes and the average GC content was 64.58%. Additionally, an average of 3.1% of sequences and 12.8% bps remained unbinned after processing (Table S2).

Eight phyla (Actinomycetota, Pseudomonodota [previously Proteobacteria], Chloroflexota, Bacteroidota, Acidobacteriota, Cyanobacteriota, Gemmatimonadota and Verrucomicrobiota) of the 20 total phyla accounted for 93% of the MAGs. The majority (55%) of MAGs belonged to the phylum Actinomycetota, while Pseudomonadota (12%) and Chloroflexota (10%) together comprised nearly half (49%) of the remainder. Five taxonomic identifications were resolved to the species level: *Pseudomonas E antarctica*, *P. E gergormendelli*, *Carnobacterium A pleistocenium, C. A alterfunditum* and *Flavobacterium fryxellicola* (data not shown). We also recovered a single archaeal MAG, an unclassified *Nitrosocosmicus* (phylum Thermoproteota, order Nitrosphaerales) that was 83.65% complete and had 2.85% contamination (data not shown).

Only a small fraction of the reads mapped to the genomes in our custom database (Figure S4). Reads mapped better to the JGI GOLD sequences (~40%) than to refseq (~28%) and surprisingly poorly to genomes from the Antarctic literature (~4%). The vast majority of reads did not map with any existing sequences, while an average of ~23,000 (0.12% of the total) reads mapped once and 14,000 (0.08%) mapped more than once (Figure S4).

### 3.2 Community Structure by Climatic Zone

All MAGs occurred in all sites, but community structure depended on site climatic zone (Figure 3). Site communities clustered into three groups (PERMANOVA; *p*‐value: 0.001, *R* ^2^: 0.58) that loosely corresponded to the site's previously determined climatic zones (Marchant and Head 2007; Thompson et al. 2020). Over half (60%) of sites determined as belonging to the coastal zone (near the coast, low elevation) clustered instead with inland or upland sites (Figure 3A). For the purposes of this paper, we reassigned zones based on Figure 3A. Diversity in coastal sites was significantly different from inland and upland sites according to both the Shannon and Simpson diversity indices (Kruskal–Wallis Nonparametric test; *p*‐value: 0.38 and 0.39, respectively; Pairwise tests: *p*‐values: 0.36 and 0.38, respectively). Clusters were dominated by distinct taxa, though there was overlap between the taxonomic profiles of the inland and upland sites while there was none between the coastal cluster communities and the inland and upland communities.

At the phylum level, community structure differed substantially between the coastal cluster and the upland and inland clusters, but was highly consistent between the inland and upland sites. An unclassified phylum (Bacteria_X), Deinococcota, Gemmatimonadota, Acidobacteriota, Actinomycetota and Chloroflexota were the most abundant phyla in the inland and upland zone communities, except for Marr Pond, Cliff Nunatak and Mt. Seuss. Conversely, the phyla Bacillota, Desulfobacteriota_F, Bacillota_A and Patescibacteria had the lowest relative abundance at all sites except Hjorth Hill, where they were the most abundant. The coastal sites were dominated by Cyanobacteria, J088, Myxococcota, JAGOEH01, Pseudomonadota and Chlamydiota (Figure 4A).

At lower taxonomic levels (i.e., family), the structure of the inland and upland clusters became distinct (but remained relatively homogenous internally) and sites in the coastal cluster showed significant differences to one another (Figure 4B). Two unnamed groups, P2‐11 E (Chloroflexota) and JACCXJ01 were consistently the most abundant families in the inland zone, except for Chroococcidiopsidaeceae (Cyanobacteriota) at Mount Suess. Egibacteriaceae, an unnamed group SCDTD01, and an unclassified Actinomycetales (all Actinomycetota) were highly abundant across the upland cluster (Figure 4B).

When considering the 25 most abundant taxa per zone, the upland and inland zones shared 16 of their top 25 taxa. However, all of the top 25 taxa from the coastal zone were unique to that zone (Figure 3C). The structure of the top 25 taxa in each zone was characterised by a change from Actinomycetota to non‐Actinomycetota bacteria; Actinomycetota were 24 of the top 25 taxa in the upland zone, 19 in the inland zone and 13 of the coastal sites. However, the 13 Actinomycetota from the coastal sites were mostly different families (Illumatobacteraceae, Propionibacteriaceae, Dermatophilaceae and Nocardioidaceae) than those found in the upland and inland zones (Solirubrobacteraceae, Thermoleophilaceae, Nocardioidaceae and Rubrobacteraceae). Nocardioidaceae and Illumatobacteraceae were the only families to occur in the top 25 taxa in all three zones, though there was no overlap between the top 25 coastal taxa and the taxa from the other zones at the species level (Figure 6). The non‐Actinomycetota in the coastal zone consisted of phyla Cyanobacteria (families Nostocaceae, Leptolyngbyaceae and Elainellaceae), Bacteroidota, Chloroflexota, J088 and Pseudomonadota.

### 3.3 Functional Ecology of MDV Soil Bacteria Communities

Overall, few taxa possessed the complete set of genes for key KEGG modules in these categories, although many possessed partially complete pathways for those modules (Figures 5B and S5). Modules contributing to interspecies competition (e.g., secondary metabolites, virulence factors, antibiotic biosynthesis and antibiotic resistance) were likewise rarely complete and also infrequently partially complete (Figures 6B and S5).

### 3.4 Nutrient Metabolism

For carbon metabolism, most taxa possessed some genes involved in each CO_2_ metabolism KEGG pathways (Calvin cycle [99.4%], Arnon–Buchanan cycle [100%], Wood–Ljungdahl pathway [71%], the hydroxypropionate–hydroxybutylate cycle [79%], 3‐Hydroxypropionate bicycle [98%] and the dicarboxylate–hydroxybutyrate cycle [98%]). However, average completion was low across phyla (Figure 5A; less than 25% completion for all pathways except the Arnon–Buchanan [41%] and Calvin [56%] cycles) and none had a 100% complete gene set for any of these pathways except four taxa for the Calvin cycle (two alphaproteobacterial [Bradyrhizobium, Sphingomonas] and two gammaproteobacteria [Dokdonella, Rhodoferax]). An additional 39 taxa had 91% complete KEGG Calvin cycle genesets. Of the other carbon fixation cycles, the Arnon–Buchanan cycle pathway was most complete across taxa, with 148 SLCs possessing ≥ 50% of the pathway's genes, 28 ≥ 70% and 3 ≥ 80% of the pathway genes (a Pseudoxanthomonas [Xanthomonodaceae, Gammaproteobacteria]; and unidentified taxa in the Dermatophilaceae [Actinomycetota] and Opitutaceae [Verrucomicrobiota]).

**FIGURE 9:**
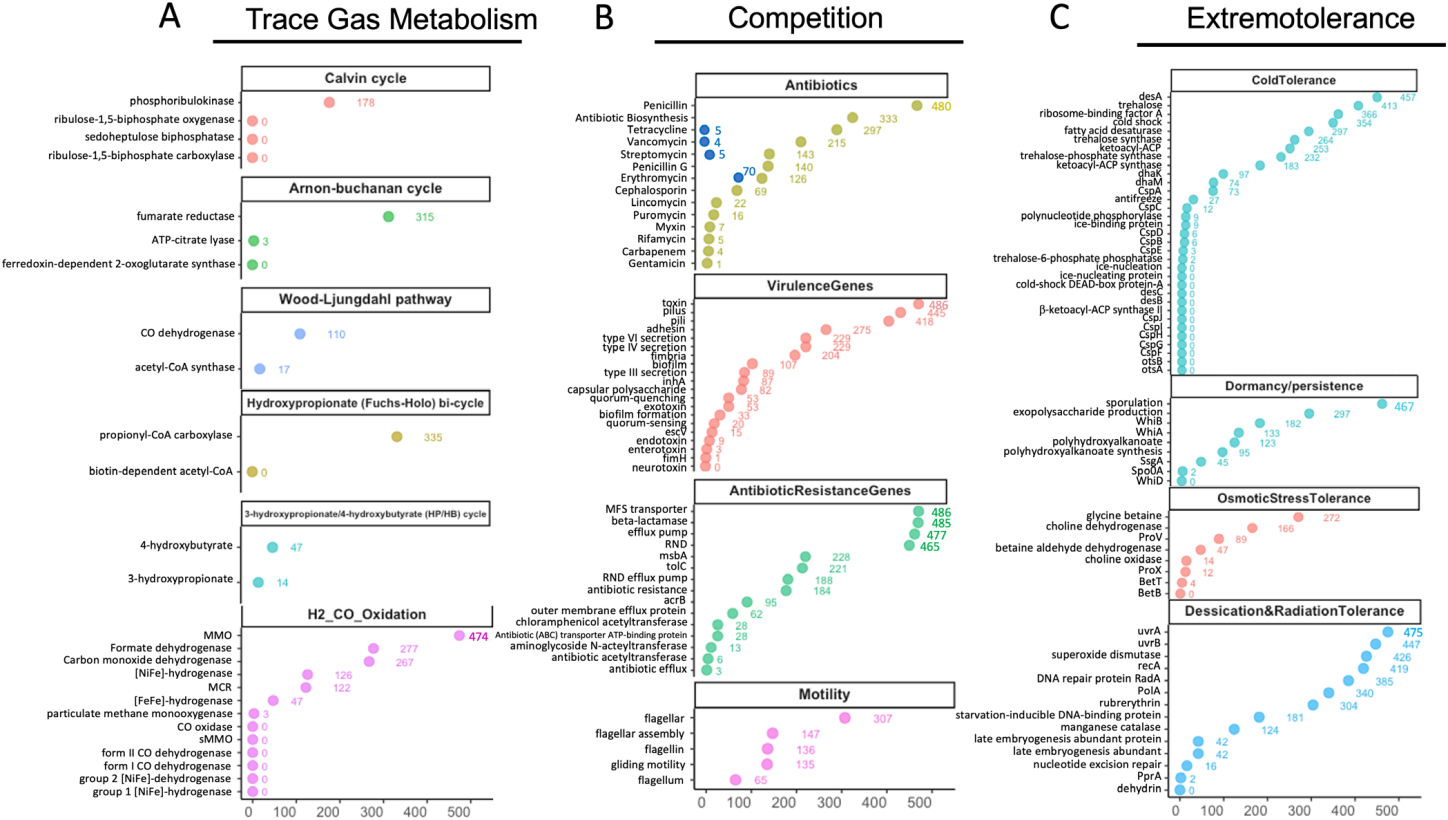
Phenotypic keyword hits by taxa. Phenotypes are organised by category, with gene keywords (e.g., antibiotic acetyltransferase) on the *y*‐axis and taxa counts on the *x*‐axis. Counts (*x*‐axis) represent unique taxa with at least 1 hit for any given phenotype/gene. Keywords with 0 hits were excluded from the figure; (A) trace gas metabolism, (B) competition and (C) extremotolerance.

We recovered hits against at least one gene considered potentially diagnostic of each cycle (Berg 2011) in all cycles except the dicarboxylate–hydroxybutyrate cycle (Figure 9A). Three cycles had key genes in a significant proportion of taxa: 178 taxa had phosphoribulokinase (Calvin cycle), 335 taxa had propionyl‐CoA carboxylase (hydroxypropionate [Fuchs–Holo] bicycle) and 315 taxa had fumarate reductase (Arnon–Buchanan cycle), though only two had taxa that possessed all enzymes associated with the cycle (the Wood–Ljungdahl pathway [17] and the 3‐hydroxypropionate/4‐hydroxybutyrate (HP/HB) cycle [14]). Additionally, a number of genes coding for CO metabolism (formate and CO dehydrogenase) and H_2_ gas oxidation ([Ni‐Fe]‐ and [FeFe]‐hydrogenase) occurred in roughly half and one quarter of the SLCs, respectively.

For nitrogen metabolism, only members of Desulfobacteriota possessed near complete KEGG pathways for nitrogen fixation, while some portions of the KEGG nitrification pathway occurred in the bacterial phyla Chlamydiota and J088 and the archaeal phylum Thermoproteota (Figure 5). Nitrate reduction and denitrification were more common across phyla but 100% completion of the pathway in KEGG was rare (Figure 5). Assimilatory nitrate reduction was more widespread among the top 25 taxa of the inland and upland zones than among the coastal (12 and 12 vs. 5, respectively). In contrast, dissimilatory nitrate reduction was more common in coastal site taxa than in the inland and upland zones (11 vs. 6 and 5, respectively; Figure 7). Nitrogen fixation and nitrification via ammonia pathway genes were absent from the top 25 taxa in the inland and upland zones, while nitrification via commamox was more common in the coastal sites than nitrification via ammonia, and rare outside the coastal zone (Figure 7).

### 3.5 Competitive Interactions

Few KEGG modules associated with competitive interactions were complete or even partially complete (Figure 6). Only beta‐lactam resistance was broadly present and mostly complete across phyla and dominant taxa in each zone (Figure 8), with more than 60 taxa possessing a 100% complete KEGG gene suite for the pathway (Figure 6B). Vancomycin resistance (D‐Ala‐D‐Lac type) and pyocyanine biosynthesis were just as widely distributed as beta‐lactam resistance, although much less complete (no taxon had a fully complete gene suite for these pathways; Figure 6). Type II polyketide backbone biosynthesis and multidrug resistance efflux pump QacA were sporadically present in phyla from all zones, and were likewise incomplete in all taxa (Figures 6B and 8). Overall, there was more competition‐associated functional diversity in coastal‐dominant taxa than there was in the upland or inland zones with genes from nine modules uniquely in the coastal zone compared to four in the upland/inland zone (Figure 8).

Our gene annotation file queries provided additional insight. We recovered hits on 13 of 77 antibiotic keywords (Table S3) in our annotation files, seven of which occurred in more than 100 MAGs (Figure 9). Of those that did occur across taxa, all but one (erythromycin) consisted of mostly hits pointing to genes involved in resistance to those compounds. Erythromycin returned more than half of its gene hits containing the string ‘erythromycin biosynthesis’. To estimate the number of distinct taxa that possessed biosynthesis genes for these widely occurring antibiotics, we added additional queries to our list combining the antibiotic's name and ‘biosynthesis’, then added the results to Figure 9B as dark blue circles.

Beyond antibiotics, genes associated with toxin production and pili were nearly ubiquitous across taxa (486 and 445, respectively), while adherence and type IV and IV secretion systems were less common but still present in roughly half of the MAGs. General antibiotic resistance phenotypes (major family system transporters, efflux pumps and RND) were widespread, while phenotypes that targeted specific antibiotics (e.g., various acetyltransferases) were rarer. Additionally, at least 28% of MAGs possessed genes associated with flagella, and an equal number with gliding motility. Slightly fewer (~20%) possessed genes containing the keyword biofilm.

### 3.6 Extremotolerance Genes

We queried four categories of extremotolerance genes (cold tolerance, dormancy/persistence, osmotic stress tolerance and desiccation and radiation tolerance) against our annotation files to ascertain which strategies MDV bacteria were using to survive in the MDVs. Cold tolerance strategies including fatty acid desaturase (and the accompanying gene desA), trehalose and cold shock associated proteins, were found in a majority (54%, 95%, 85% and 61%, respectively) of MAGs, while sporulation and exopolysaccharide production were common (96% and 61%, respectively) dormancy strategies. Over half of MDV taxa also possessed glycine betaine and choline dehydrogenase genes, both involved in the production and export of the osmoprotectant glycine betaine. Genes and phenotypes typically associated with DNA damage repair (uvrAB, recA), and reactive oxygen species scavenging (superoxide dismutase) were also nearly ubiquitous strategies for managing the effects of desiccation and irradiation.

## 4 Discussion

### 4.1 Taxonomic Diversity (MAGs Diversity and Assembly Completeness)

Our results reveal a diverse and previously under‐characterised bacterial community in MDV soils, with 701 medium‐to‐high quality MAGs corresponding to an estimated 484 unique species‐level genomes spanning distinct environmental gradients. This is 55% more MAGs than previously recovered in a study of similar sites (Ortiz et al. 2021), suggesting that microbial diversity in polar desert soils exceeds that previously recognised. Though this discrepancy in MAG count between studies could be an artefact from the differences in sequencing depth, sampling breadth and binning approaches (Vosloo et al. 2021), our study used VEBA, a pipeline that uses a novel iterative prokaryote binning procedure that tends to recover additional MAGs from sites previously analysed with other approaches (Espinoza and Dupont 2022). Actinomycetota dominate this ecosystem, consistent with previous observations of Antarctic sites (Cary et al. 2010; Ji et al. 2017). Two MAGs belonged to phyla that GTDB‐tk was unable to classify. Both were from an environmentally extreme high elevation valley that has been used for astrobiology research (Goordial et al. 2016) and both were near the lower threshold for medium quality assemblies (~51% completeness and > 6% contamination), suggesting the presence of novel yet rare extremophile lineages in the more extreme MDV soils. One of the most abundant families in the upland zone (SCTD01) has also been reported from fire‐combusted soils in temperate climates (Nelson et al. 2022).

Though outside the scope of our study, we only recovered a single archaeon MAG, the nitrogen‐oxidising archaeon *Nitrosocosmicus* from the order Nitrososphaerales and phylum Thermoproteota (Alves et al. 2019). Our approach (whole environment DNA extraction and VEBA processing) does not intentionally exclude archaea, so this low recovery rate is likely due to the overall lower abundance and diversity of archaea in soils generally (Wang et al. 2020) and in Antarctic soils specifically (Lambrechts et al. 2019; Ortiz et al. 2021; Dragone et al. 2022). Methodological biases such as poorer representation in reference databases (Schloss et al. 2016; Makarova et al. 2019; Wu et al. 2025) also make archaean recovery and assembly by VEBA less likely than the better represented bacteria. This limited recovery likely reflects both their low abundance in Antarctic soils and poorer representation in reference databases, making the recovered *Nitrosocosmicus* especially significant given its wide distribution in this region's soils (Ortiz et al. 2021; Dragone et al. 2022) and its central role in nitrogen cycling (Leininger et al. 2006). Future exploration of this widespread archaeon's functioning, distribution and limitations will be critical for understanding the MDV soil nitrogen cycle (Magalhães et al. 2014) and its sensitivity to future climatic changes.

Few MAGs were resolved to genus or species level, few KEGG pathways for key metabolic and cell maintenance processes were complete, and few of our reads mapped well to genomic sequences in standard databases. Low read mapping, pathway completion and infrequent resolution to lower taxonomic levels could be due to low sequencing depth, methodological (i.e., tool) biases, incomplete reference databases, or high levels of novel genetic diversity in the sample organisms. We used standard sampling, DNA extraction and sequencing protocols (Thompson et al. 2020) and produced an average of 15 million reads per sample, enough to identify taxa although likely too low to recover gene diversity comprehensively given the read length and taxonomic complexity of the samples (Gweon et al. 2019). An estimated maximum genome diversity of ~500 species per site (this study) and a minimum of ~250 species per site (Ortiz et al. 2021; Dragone et al. 2022) results in low to moderate coverage (~3.8 to 7.5×) per genome. However, the software we used for assembly and annotation, VEBA, is relatively new and utilises a comprehensive suite of established tools that perform well at recovering bacterial MAGs, even from complex environmental samples like soil (Espinoza and Dupont 2022; Espinoza et al. 2024). Also, despite two thirds (62%) of our MAGs being less than 80% complete, 17% (84/481) of the species‐level clusters (SLCs) had two or more MAGs assigned to them, a redundancy that would have captured more of the genome than a single MAG could. Moreover, as a reference database, KEGG is ideal for well‐studied organisms (e.g., clinical taxa) but is less predictive for poorly studied taxa, like those in polar soils (Albright and Louca 2023). Indeed, our sequences also mapped poorly to a custom database we compiled which included environmentally sourced genomes as well as the highly curated refseq (Figure S4). Rather than solely reflecting technical limitations, the consistent partial completion of multiple pathways suggests Antarctic bacteria may employ novel or hybridised metabolic strategies not yet captured in KEGG.

A high degree of genetic novelty would not be surprising as Antarctic soil communities are highly isolated both geographically and temporally (Convey et al. 2008, 2009), Antarctic bacteria are genetically distinct at fine taxonomic scales (Albanese et al. 2021), and standard databases represent environmental isolates poorly, particularly from less studied environments (McGee et al. 2019). Our results support the notion that further exploration of Antarctic soil bacterial genomes and phenotypes will likely yield previously unknown metabolic pathways and survival strategies (Rizzo et al. 2021; Waschulin et al. 2022). Such knowledge can provide insights into ecological genomics (Xue et al. 2024), applied microbiology (Mesbah 2022), human health (Núñez‐Montero and Barrientos 2018), and astrobiology (Rawat et al. 2024).

### 4.2 Community Structure and Functional Ecology

MDV soils appear to be dominated by two general communities with distinct, albeit related, functional profiles, correlating to long‐term moist areas (e.g., coastal sites) and long‐term arid sites (i.e., inland and upland sites). The long‐term arid sites are dominated by gram‐positive Actinomycetota and possess a more homogenous functional profile, while the long‐term moist sites possess greater phylogenetic and functional diversity, but these patterns are likely to shift with climate change.

### 4.3 Nutrient Metabolism

We found widespread evidence for trace gas metabolism as a key survival strategy. A substantial number of MDV bacteria possess key genes for fixing atmospheric carbon non‐photosynthetically via the Calvin and Arnon–Buchanan cycles, and the hydroxypropionate (Fuchs‐Holo) bicycle. While many taxa contained genes for multiple carbon fixation pathways, few possessed fully complete KEGG pathways, suggesting that mix‐and‐match metabolic flexibility may be common in these resource‐limited soils (Ortiz et al. 2021). These results align with recent studies emphasising the role of atmospheric trace gas oxidation in polar desert microbiomes (Ji et al. 2017; Ortiz et al. 2021) and soils globally (Bay et al. 2021) and confirm previous inferences (e.g., Ortiz et al. 2021) with higher taxonomic resolution.

We confirm that nitrogen fixation does not appear to occur outside moist sites (Cary et al. 2010), although taxa capable of performing assimilatory nitrate reduction are more common in arid soils than in moist sites.

### 4.4 Competitive Interactions and Extremotolerance

The broad occurrence of antibiotic synthesis and resistance genes in our MAGs suggests that antibiotic‐mediated interactions are a likely factor in MDV community dynamics. Further, few taxa possessed genes associated with common antibiotics (Table S3), including tetracycline, vancomycin and streptomycin, which suggests the presence of potentially novel antibiotic compounds in Antarctic soil bacteria (Núñez‐Montero and Barrientos 2018; Quinn and Dyson 2024).

The presence of antibiotic‐resistance genes in natural environments often indicates contamination from human sources (Ondon et al. 2021), but our samples were unlikely to be contaminated in this way. MDV soils are among the most pristine soils in the world due to their historical isolation, relative inaccessibility and current management policies (Hwengwere et al. 2022); many of our study sites are rarely visited even by Antarctic soil scientists (e.g., sites west and north of Taylor Valley, see Figure 1), DNA extraction was performed using sterile technique (Thompson et al. 2020), and our MAG composition parallels that found in other Antarctic soils (Ortiz et al. 2021; Dragone et al. 2022). In reality, both antibiotic production and resistance are natural attributes of soil communities globally (Spagnolo et al. 2021), which use these compounds for a variety of roles, including antagonism and cell‐to‐cell signalling (Davies 2006; Spagnolo et al. 2021). Of course, the presence of genes in a genome does not indicate that they are expressed, or how often. However, Antarctic soil microbes have likely been isolated from ancestral lineages for millions of years (Convey et al. 2008, 2009) and could have easily lost these genes since then if they conferred no advantage in the Antarctic environment, especially considering its extreme selective pressures (Mira et al. 2001; Koskiniemi et al. 2012). Thus, the widespread occurrence of these genes in our MAGs suggests that they play a role in community dynamics in this ecosystem. Though this observation may seem to conflict with previous observations that the MDVs are dominated by abiotic drivers (Hogg et al. 2006; Lee et al. 2019; Lebre et al. 2023), previous work has suggested that extreme abiotic factors may actually encourage the use of antibiotics in polar environments by increasing competition for scarce resources among fewer competitors (Pantůček et al. 2018). Future research should explore the rate of expression of these compounds (if any), their potential targets, and how these interactions might map to MDV community interaction networks (Thompson et al. 2021).

Our data also confirm that MDV bacteria invest heavily in genetic and physiological extreme tolerance survival mechanisms, suggesting that selection for taxa that can manage both biotic and abiotic pressures must be high. Many MAGs possessed genes predicted to be involved in managing low temperatures (fatty acid desaturase), long‐term unfavourable conditions (sporulation), ice nucleation (glycine betaine, trehalose and exopolysaccharide production) and DNA damage repair and ROS scavenging (uvrAB and superoxide dismutase). Few MAGs possessed the genes often associated with these cellular processes (e.g., otsAB for trehalose synthase, CSPA‐J for the cold shock response) (Lim et al. 2019; Zhang and Gross 2021; Scales et al. 2023), again suggesting that MDV bacteria are utilising novel gene pathways for key survival phenotypes. However, this observation could also be the result of non‐standard gene naming in databases, erroneous assemblies, or other limitations of predictive gene annotation using standard reference databases (Salzberg 2019).

Finally, many taxa possessed genes for motility features, such as flagella and gliding motility, as well as genes with the keyword ‘biofilm’. However, these phenotypes were found in only a subset of MAGs, suggesting that MDV bacteria lifestyles are diverse and that the soils they inhabit are likely to contain several micro‐niches (Vos et al. 2013; Larkin and Martiny 2017; Baquero et al. 2021).

## 5 Conclusions

In conclusion, we recovered 50% more medium‐ and high‐quality MAGs than previously reported for similar Antarctic sites. We also report MAGs from sites in the same features as those studied as part of the MCM LTER, expanding the known genomic diversity and multifunctionality of these ecosystems. Our findings challenge the appropriateness of using previously designated climatic zones as proxies for microbial community structure, suggesting that localised abiotic and biotic factors may play a larger role in shaping microbial assemblages. However, we suggest that community composition remains distinct between moist, vegetated sites and arid, regolith‐based sites. We provide strong evidence for diverse carbon fixation strategies in MDV taxa, extending beyond photosynthetically driven carbon fixation and spanning multiple climatic zones. Our data suggest that interspecies competition, particularly antibiotic‐mediated interactions, may be a significant structuring force in microbial food web dynamics in these soils. While abiotic controls remain the dominant driver of MDV community structure, the broad distribution of antibiotic resistance and synthesis genes indicates that antibiotic‐mediated interactions may have increased influence under resource‐limited or transiently favourable conditions. Finally, our results support previous findings that Antarctic bacteria likely possess numerous novel genes and pathways involved in key nutrient metabolism pathways, extremotolerance and antibiotic biosynthesis. These insights underscore the potential for unique microbial adaptations in extreme polar desert environments and highlight the need for further exploration of their functional roles in Antarctic ecosystems.

## Author Contributions

**A. R. Thompson:** conceptualization, writing – original draft, writing – review and editing, visualization, validation, methodology, investigation, formal analysis, software, data curation. **S. Yooseph:** supervision, writing – review and editing, conceptualization, funding acquisition, project administration, resources. **B. J. Adams:** funding acquisition, writing – review and editing, resources. **I. D. Hogg:** funding acquisition, writing – review and editing, resources.

## Funding

This work was supported by the National Science Foundation (NSF) Grant numbers ANT 2133685 and OPP‐2224760 to B.J. Adams and is a contribution to the McMurdo Dry Valleys Long‐Term Ecological Research (LTER) programme. Antarctica New Zealand and the New Zealand Antarctic Research Institute (NZARI) provided logistics and financial support through Event K024 to I.D. Hogg. ART and SY were supported by NSF grants #DBI‐2400009 and #OAC‐2408259. Geospatial support for this work was provided by the Polar Geospatial Center under NSF‐OPP awards 1043681, 1559691 and 2129685.

## Conflicts of Interest

The authors declare no conflicts of interest.

## Supporting information


**Figure S1:** MAG and SLC genome size (est. gene count).
**Figure S2:** Metadata overview.
**Figure S3:** Alignment success and protein divergence.*
**Figure S4:** KEGG completion ratio boxplots.
**Figure S5:** Taxon relative abundance distribution.
**Table S1:** Sequencing stats.
**Table S2:** Phenotype keywords.
**Table S3:** Sample physicochemical and climatic zone metadata.
**Table S4:** Alpha and beta diversity stats.

**Data S1:** Supporting Information.

## Data Availability

The data that support the findings of this study are openly available in WGS 18MDV 2018 at https://www.ncbi.nlm.nih.gov/bioproject/?term=WGS+18MDV+2018, reference number PRJNA1251696.

## References

[emi470249-bib-0001] Adams, B. J. , R. D. Bardgett , E. Ayres , et al. 2006. “Diversity and Distribution of Victoria Land Biota.” Soil Biology and Biochemistry 38: 3003–3018.

[emi470249-bib-0002] Adriaenssens, E. M. , R. Kramer , M. W. Van Goethem , T. P. Makhalanyane , I. Hogg , and D. A. Cowan . 2017. “Environmental Drivers of Viral Community Composition in Antarctic Soils Identified by Viromics.” Microbiome 5: 83. 28724405 10.1186/s40168-017-0301-7 PMC5518109

[emi470249-bib-0003] Albanese, D. , C. Coleine , O. Rota‐Stabelli , et al. 2021. “Pre‐Cambrian Roots of Novel Antarctic Cryptoendolithic Bacterial Lineages.” Microbiome 9: 63. 33741058 10.1186/s40168-021-01021-0 PMC7980648

[emi470249-bib-0004] Albright, S. , and S. Louca . 2023. “Trait Biases in Microbial Reference Genomes.” Scientific Data 10: 84. 36759614 10.1038/s41597-023-01994-7 PMC9911409

[emi470249-bib-0005] Alneberg, J. , B. S. Bjarnason , I. de Bruijn , et al. 2014. “Binning Metagenomic Contigs by Coverage and Composition.” Nature Methods 11: 1144–1146. 25218180 10.1038/nmeth.3103

[emi470249-bib-0006] Alves, R. J. E. , M. Kerou , A. Zappe , et al. 2019. “Ammonia Oxidation by the Arctic Terrestrial Thaumarchaeote Candidatus *Nitrosocosmicus arcticus* Is Stimulated by Increasing Temperatures.” Frontiers in Microbiology 10: 1571. 31379764 10.3389/fmicb.2019.01571 PMC6657660

[emi470249-bib-0007] Andriuzzi, W. S. , B. J. Adams , J. E. Barrett , R. A. Virginia , and D. H. Wall . 2018. “Observed Trends of Soil Fauna in the Antarctic Dry Valleys: Early Signs of Shifts Predicted Under Climate Change.” Ecology 99: 312–321. 29315515 10.1002/ecy.2090

[emi470249-bib-0009] Aramaki, T. , R. Blanc‐Mathieu , H. Endo , et al. 2019. “MetaBAT 2: An Adaptive Binning Algorithm for Robust and Efficient Genome Reconstruction From Metagenome Assemblies.” PeerJ 7: e7359. 31388474 10.7717/peerj.7359 PMC6662567

[emi470249-bib-0008] Aramaki, T. , R. Blanc‐Mathieu , H. Endo , et al. 2020. “KofamKOALA: KEGG Ortholog Assignment Based on Profile HMM and Adaptive Score Threshold.” Bioinformatics 36: 2251–2252. 31742321 10.1093/bioinformatics/btz859 PMC7141845

[emi470249-bib-0010] Aroney, S. T. N. , R. J. P. Newell , J. Nissen , A. P. Camargo , G. W. Tyson , and B. J. Woodcroft . 2024. “CoverM: Read Coverage Calculator for Metagenomics.” https://github.com/wwood/CoverM. 10.1093/bioinformatics/btaf147 PMC11993303 40193404

[emi470249-bib-0011] Bamforth, S. S. , D. H. Wall , and R. A. Virginia . 2005. “Distribution and Diversity of Soil Protozoa in the McMurdo Dry Valleys of Antarctica.” Polar Biology 28: 756–762.

[emi470249-bib-0012] Baquero, F. , T. M. Coque , J. C. Galán , and J. L. Martinez . 2021. “The Origin of Niches and Species in the Bacterial World.” Frontiers in Microbiology 12: 657986. 33815348 10.3389/fmicb.2021.657986 PMC8010147

[emi470249-bib-0013] Barrett, J. E. , R. A. Virginia , D. W. Hopkins , et al. 2006. “Terrestrial Ecosystem Processes of Victoria Land, Antarctica.” Soil Biology and Biochemistry 38: 3019–3034.

[emi470249-bib-0014] Bay, S. K. , X. Dong , J. A. Bradley , et al. 2021. “Trace Gas Oxidizers Are Widespread and Active Members of Soil Microbial Communities.” Nature Microbiology 6: 246–256. 10.1038/s41564-020-00811-w 33398096

[emi470249-bib-0015] Beet, C. R. , I. D. Hogg , G. E. Collins , D. A. Cowan , D. H. Wall , and B. J. Adams . 2016. “Genetic Diversity Among Populations of Antarctic Springtails (Collembola) Within the Mackay Glacier Ecotone.” Genome 59: 762–770. 27463035 10.1139/gen-2015-0194

[emi470249-bib-0016] Berg, I. A. 2011. “Ecological Aspects of the Distribution of Different Autotrophic CO_2_ Fixation Pathways.” Applied and Environmental Microbiology 77: 1925–1936. 21216907 10.1128/AEM.02473-10 PMC3067309

[emi470249-bib-0017] Bezuidt, O. K. I. , P. H. Lebre , R. Pierneef , et al. 2020. “Phages Actively Challenge Niche Communities in Antarctic Soils.” mSystems 5: e00234‐20. 32371471 10.1128/mSystems.00234-20 PMC7205518

[emi470249-bib-0018] Bolger, A. M. , M. Lohse , and B. Usadel . 2014. “Trimmomatic: A Flexible Trimmer for Illumina Sequence Data.” Bioinformatics 30: 2114–2120. 24695404 10.1093/bioinformatics/btu170 PMC4103590

[emi470249-bib-0019] Brunson, J. C. , and Q. D. Read . 2023. “ggalluvial: Alluvial Plots in ‘ggplot2’.” https://corybrunson.github.io/ggalluvial/.

[emi470249-bib-0020] Buchfink, B. , C. Xie , and D. H. Huson . 2015. “Fast and Sensitive Protein Alignment Using DIAMOND.” Nature Methods 12: 59–60. 25402007 10.1038/nmeth.3176

[emi470249-bib-0021] Buelow, H. N. , A. S. Winter , D. J. Van Horn , et al. 2016. “Microbial Community Responses to Increased Water and Organic Matter in the Arid Soils of the McMurdo Dry Valleys, Antarctica.” Frontiers in Microbiology 7: 1040. 27486436 10.3389/fmicb.2016.01040 PMC4947590

[emi470249-bib-0022] Burkins, M. B. , R. A. Virginia , and D. H. Wall . 2001. “Organic Carbon Cycling in Taylor Valley, Antarctica: Quantifying Soil Reservoirs and Soil Respiration.” Global Change Biology 7: 113–125.

[emi470249-bib-0023] Caruso, T. , I. D. Hogg , U. N. Nielsen , et al. 2019. “Nematodes in a Polar Desert Reveal the Relative Role of Biotic Interactions in the Coexistence of Soil Animals.” Communications Biology 2: 63. 30793042 10.1038/s42003-018-0260-y PMC6377602

[emi470249-bib-0024] Cary, S. C. , I. R. McDonald , J. E. Barrett , and D. A. Cowan . 2010. “On the Rocks: The Microbiology of Antarctic Dry Valley Soils.” Nature Reviews. Microbiology 8: 129–138. 20075927 10.1038/nrmicro2281

[emi470249-bib-0025] Chandra, N. , and S. Kumar . 2017. “Antibiotics Producing Soil Microorganisms.” In Antibiotics and Antibiotics Resistance Genes in Soils. Soil Biology, edited by, M. Hashmi , V. Strezov , and A. Varma , vol. 51. Springer.

[emi470249-bib-0026] Chaumeil, P.‐A. , A. J. Mussig , P. Hugenholtz , and D. H. Parks . 2020. “GTDB‐Tk: A Toolkit to Classify Genomes With the Genome Taxonomy Database.” Bioinformatics 36: 1925–1927. 10.1093/bioinformatics/btz848 PMC7703759 31730192

[emi470249-bib-0027] Chen, S. , Y. Zhou , Y. Chen , and J. Gu . 2018. “fastp: An Ultra‐Fast All‐In‐One FASTQ Preprocessor.” Bioinformatics 34: i884–i890. 30423086 10.1093/bioinformatics/bty560 PMC6129281

[emi470249-bib-0028] Collins, G. E. , and I. D. Hogg . 2016. “Temperature‐Related Activity of *Gomphiocephalus hodgsoni* (Collembola) Mitochondrial DNA (COI) Haplotypes in Taylor Valley, Antarctica.” Polar Biology 39: 379–389.

[emi470249-bib-0029] Convey, P. , J. A. Gibson , C. D. Hillenbrand , et al. 2008. “Antarctic Terrestrial Life–Challenging the History of the Frozen Continent?” Biological Reviews of the Cambridge Philosophical Society 83: 103–117. 18429764 10.1111/j.1469-185X.2008.00034.x

[emi470249-bib-0030] Convey, P. , M. I. Stevens , D. A. Hodgson , et al. 2009. “Exploring Biological Constraints on the Glacial History of Antarctica.” Quaternary Science Reviews 28: 3035–3048.

[emi470249-bib-0031] Courtright, E. M. , D. H. Wall , and R. A. Virginia . 2001. “Determining Habitat Suitability for Soil Invertebrates in an Extreme Environment: The McMurdo Dry Valleys, Antarctica.” Antarctic Science 13: 9–17.

[emi470249-bib-0032] Cycoń, M. , A. Mrozik , and Z. Piotrowska‐Seget . 2019. “Antibiotics in the Soil Environment‐Degradation and Their Impact on Microbial Activity and Diversity.” Frontiers in Microbiology 10: 338. 30906284 10.3389/fmicb.2019.00338 PMC6418018

[emi470249-bib-0033] Davies, J. 2006. “Are Antibiotics Naturally Antibiotics?” Journal of Industrial Microbiology & Biotechnology 33: 496–499. 16552582 10.1007/s10295-006-0112-5

[emi470249-bib-0034] Doran, P. T. , W. B. Lyons , and D. M. McKnight . 2010. Life in Antarctic Deserts and Other Cold Dry Environments: Astrobiological Analogs, edited by P. T. Doran , W. B. Lyons , and D. M. McKnight . Cambridge University Press.

[emi470249-bib-0035] Doran, P. T. , C. P. McKay , G. D. Clow , et al. 2002. “Valley Floor Climate Observations From the McMurdo Dry Valleys, Antarctica, 1986–2000.” Journal of Geophysical Research: Atmospheres 107: ACL‐13.

[emi470249-bib-0036] Dragone, N. B. , J. B. Henley , H. Holland‐Moritz , et al. 2022. “Elevational Constraints on the Composition and Genomic Attributes of Microbial Communities in Antarctic Soils.” Msystems 7: e0133021. 35040702 10.1128/msystems.01330-21 PMC8765064

[emi470249-bib-0037] Espinoza, J. L. , and C. L. Dupont . 2022. “VEBA: A Modular End‐To‐End Suite for In Silico Recovery, Clustering, and Analysis of Prokaryotic, Microeukaryotic, and Viral Genomes From Metagenomes.” BMC Bioinformatics 23: 419. 36224545 10.1186/s12859-022-04973-8 PMC9554839

[emi470249-bib-0038] Espinoza, J. L. , A. Phillips , M. B. Prentice , et al. 2024. “Unveiling the Microbial Realm With VEBA 2.0: A Modular Bioinformatics Suite for End‐To‐End Genome‐Resolved Prokaryotic, (Micro)eukaryotic and Viral Multi‐Omics From Either Short‐ or Long‐Read Sequencing.” Nucleic Acids Research 52: e63. 38909293 10.1093/nar/gkae528 PMC11317156

[emi470249-bib-0039] Fell, J. W. , G. Scorzetti , L. Connell , and S. Craig . 2006. “Biodiversity of Micro‐Eukaryotes in Antarctic Dry Valley Soils With <5% Soil Moisture.” Soil Biology and Biochemistry 38: 3107–3119.

[emi470249-bib-0040] Fierer, N. , J. W. Leff , B. J. Adams , et al. 2012. “Cross‐Biome Metagenomic Analyses of Soil Microbial Communities and Their Functional Attributes.” Proceedings of the National Academy of Sciences of the United States of America 109: 21390–21395. 23236140 10.1073/pnas.1215210110 PMC3535587

[emi470249-bib-0041] Fountain, A. G. , J. S. Levy , M. N. Gooseff , and D. Van Horn . 2014. “The McMurdo Dry Valleys: A Landscape on the Threshold of Change.” Geomorphology 225: 25–35.

[emi470249-bib-0042] Gokul, J. K. , A. Valverde , M. Tuffin , S. C. Cary , and D. A. Cowan . 2013. “Micro‐Eukaryotic Diversity in Hypolithons From Miers Valley, Antarctica.” Biology 2: 331–340. 24832664 10.3390/biology2010331 PMC4009862

[emi470249-bib-0043] Goordial, J. , A. Davila , D. Lacelle , et al. 2016. “Nearing the Cold‐Arid Limits of Microbial Life in Permafrost of an Upper Dry Valley, Antarctica.” ISME Journal 10: 1613–1624. 27323892 10.1038/ismej.2015.239 PMC4918446

[emi470249-bib-0044] Gooseff, M. N. , J. E. Barrett , B. J. Adams , et al. 2017. “Decadal Ecosystem Response to an Anomalous Melt Season in a Polar Desert in Antarctica.” Nature Ecology & Evolution 1: 1334–1338. 29046542 10.1038/s41559-017-0253-0

[emi470249-bib-0045] Gweon, H. S. , L. P. Shaw , J. Swann , et al. 2019. “The Impact of Sequencing Depth on the Inferred Taxonomic Composition and AMR Gene Content of Metagenomic Samples.” Environmental Microbiomes 14: 7. 10.1186/s40793-019-0347-1 PMC8204541 33902704

[emi470249-bib-0046] Hogg, I. D. , S. C. Cary , P. Convey , et al. 2006. “Biotic Interactions in Antarctic Terrestial Ecosystems – Are They a Factor?” Soil Biology & Biochemistry 38: 3035–3040.

[emi470249-bib-0047] Hwengwere, K. , H. Paramel Nair , K. A. Hughes , L. S. Peck , M. S. Clark , and C. A. Walker . 2022. “Antimicrobial Resistance in Antarctica: Is It Still a Pristine Environment?” Microbiome 10: 71. 35524279 10.1186/s40168-022-01250-x PMC9072757

[emi470249-bib-0048] Hyatt, D. , G.‐L. Chen , P. F. LoCascio , M. L. Land , F. W. Larimer , and L. J. Hauser . 2010. “Prodigal: Prokaryotic Gene Recognition and Translation Initiation Site Identification.” BMC Bioinformatics 11: 119. 20211023 10.1186/1471-2105-11-119 PMC2848648

[emi470249-bib-0049] Jain, C. , L. M. Rodriguez‐R , A. M. Phillippy , K. T. Konstantinidis , and S. Aluru . 2018. “High Throughput ANI Analysis of 90K Prokaryotic Genomes Reveals Clear Species Boundaries.” Nature Communications 9: 5114. 10.1038/s41467-018-07641-9 PMC6269478 30504855

[emi470249-bib-0050] Ji, M. , C. Greening , I. Vanwonterghem , et al. 2017. “Atmospheric Trace Gases Support Primary Production in Antarctic Desert Surface Soil.” Nature 552: 400–403. 29211716 10.1038/nature25014

[emi470249-bib-0051] Jones, M. B. , S. K. Highlander , E. L. Anderson , et al. 2015. “Library Preparation Methodology Can Influence Genomic and Functional Predictions in Human Microbiome Research.” Proceedings of the National Academy of Sciences of the United States of America 112: 14024–14029. 26512100 10.1073/pnas.1519288112 PMC4653211

[emi470249-bib-0052] Karlicki, M. , S. Antonowicz , and A. Karnkowska . 2022. “Tiara: Deep Learning‐Based Classification System for Eukaryotic Sequences.” Bioinformatics 38: 344–350. 34570171 10.1093/bioinformatics/btab672 PMC8722755

[emi470249-bib-0053] Kolde, R. 2018. “pheatmap: Pretty Heatmaps.” https://github.com/raivokolde/pheatmap.

[emi470249-bib-0054] Koskiniemi, S. , S. Sun , O. G. Berg , and D. I. Andersson . 2012. “Selection‐Driven Gene Loss in Bacteria.” PLoS Genetics 8: e1002787. 22761588 10.1371/journal.pgen.1002787 PMC3386194

[emi470249-bib-0055] Lambrechts, S. , A. Willems , and G. Tahon . 2019. “Uncovering the Uncultivated Majority in Antarctic Soils: Toward a Synergistic Approach.” Frontiers in Microbiology 10: 242. 30828325 10.3389/fmicb.2019.00242 PMC6385771

[emi470249-bib-0056] Langmead, B. , and S. L. Salzberg . 2012. “Fast Gapped‐Read Alignment With Bowtie 2.” Nature Methods 9: 357–359. 22388286 10.1038/nmeth.1923 PMC3322381

[emi470249-bib-0057] Larkin, A. A. , and A. C. Martiny . 2017. “Microdiversity Shapes the Traits, Niche Space, and Biogeography of Microbial Taxa.” Environmental Microbiology Reports 9: 55–70. 28185400 10.1111/1758-2229.12523

[emi470249-bib-0058] Lebre, P. H. , J. Bosch , C. Coclet , et al. 2023. “Expanding Antarctic Biogeography: Microbial Ecology of Antarctic Island Soils.” Ecography 2023: e06568.

[emi470249-bib-0059] Lee, C. K. , B. A. Barbier , E. M. Bottos , I. R. McDonald , and S. C. Cary . 2012. “The Inter‐Valley Soil Comparative Survey: The Ecology of Dry Valley Edaphic Microbial Communities.” ISME Journal 6: 1046–1057. 22170424 10.1038/ismej.2011.170 PMC3329106

[emi470249-bib-0060] Lee, C. K. , D. C. Laughlin , E. M. Bottos , et al. 2019. “Biotic Interactions Are an Unexpected Yet Critical Control on the Complexity of an Abiotically Driven Polar Ecosystem.” Communications Biology 2: 62. 30793041 10.1038/s42003-018-0274-5 PMC6377621

[emi470249-bib-0061] Leininger, S. , T. Urich , M. Schloter , et al. 2006. “Archaea Predominate Among Ammonia‐Oxidizing Prokaryotes in Soils.” Nature 442: 806–809. 16915287 10.1038/nature04983

[emi470249-bib-0062] Lim, S. , J.‐H. Jung , L. Blanchard , and A. de Groot . 2019. “Conservation and Diversity of Radiation and Oxidative Stress Resistance Mechanisms in Deinococcus Species.” FEMS Microbiology Reviews 43: 19–52. 30339218 10.1093/femsre/fuy037 PMC6300522

[emi470249-bib-0063] Loftus, M. , S. A.‐D. Hassouneh , and S. Yooseph . 2021. “Bacterial Associations in the Healthy Human Gut Microbiome Across Populations.” Scientific Reports 11: 2828. 33531651 10.1038/s41598-021-82449-0 PMC7854710

[emi470249-bib-0064] Magalhães, C. M. , A. Machado , B. Frank‐Fahle , C. K. Lee , and S. C. Cary . 2014. “The Ecological Dichotomy of Ammonia‐Oxidizing Archaea and Bacteria in the Hyper‐Arid Soils of the Antarctic Dry Valleys.” Frontiers in Microbiology 5: 515–515. 25324835 10.3389/fmicb.2014.00515 PMC4179728

[emi470249-bib-0065] Makarova, K. S. , Y. I. Wolf , and E. V. Koonin . 2019. “Towards Functional Characterization of Archaeal Genomic Dark Matter.” Biochemical Society Transactions 47: 389–398. 30710061 10.1042/BST20180560 PMC6393860

[emi470249-bib-0066] Marchant, D. R. , and J. W. Head . 2007. “Antarctic Dry Valleys: Microclimate Zonation, Variable Geomorphic Processes, and Implications for Assessing Climate Change on Mars.” Icarus 192: 187–222.

[emi470249-bib-0067] Mashamaite, L. , P. H. Lebre , G. Varliero , et al. 2023. “Microbial Diversity in Antarctic Dry Valley Soils Across an Altitudinal Gradient.” Frontiers in Microbiology 14: 1203216. 37555066 10.3389/fmicb.2023.1203216 PMC10406297

[emi470249-bib-0068] McGee, K. M. , C. V. Robinson , and M. Hajibabaei . 2019. “Gaps in DNA‐Based Biomonitoring Across the Globe.” Frontiers in Ecology and Evolution 7.

[emi470249-bib-0069] Mesbah, N. M. 2022. “Industrial Biotechnology Based on Enzymes From Extreme Environments.” Frontiers in Bioengineering and Biotechnology 10: 870083. 35480975 10.3389/fbioe.2022.870083 PMC9036996

[emi470249-bib-0070] Mira, A. , H. Ochman , and N. A. Moran . 2001. “Deletional Bias and the Evolution of Bacterial Genomes.” Trends in Genetics 17: 589–596. 11585665 10.1016/s0168-9525(01)02447-7

[emi470249-bib-0071] Mistry, J. , S. Chuguransky , L. Williams , et al. 2021. “Pfam: The Protein Families Database in 2021.” Nucleic Acids Research 49: D412–D419. 33125078 10.1093/nar/gkaa913 PMC7779014

[emi470249-bib-0072] Mistry, J. , R. D. Finn , S. R. Eddy , A. Bateman , and M. Punta . 2013. “Challenges in Homology Search: HMMER3 and Convergent Evolution of Coiled‐Coil Regions.” Nucleic Acids Research 41: e121. 23598997 10.1093/nar/gkt263 PMC3695513

[emi470249-bib-0073] Monteiro, M. , M. S Baptista , J. Séneca , et al. 2020. “Understanding the Response of Nitrifying Communities to Disturbance in the McMurdo Dry Valleys, Antarctica.” Microorganisms 8: 404. 32183078 10.3390/microorganisms8030404 PMC7143839

[emi470249-bib-0074] Mukherjee, S. , D. Stamatis , C. T. Li , et al. 2025. “Genomes OnLine Database (GOLD) v.10: New Features and Updates.” Nucleic Acids Research 53: D989–D997. 39498478 10.1093/nar/gkae1000 PMC11701667

[emi470249-bib-0075] Nearing, J. T. , G. M. Douglas , M. G. Hayes , et al. 2022. “Microbiome Differential Abundance Methods Produce Different Results Across 38 Datasets.” Nature Communications 13: 342. 10.1038/s41467-022-28034-z PMC8763921 35039521

[emi470249-bib-0076] Nelson, A. R. , A. B. Narrowe , C. C. Rhoades , et al. 2022. “Wildfire‐Dependent Changes in Soil Microbiome Diversity and Function.” Nature Microbiology 7: 1419–1430. 10.1038/s41564-022-01203-y PMC9418001 36008619

[emi470249-bib-0077] Núñez‐Montero, K. , and L. Barrientos . 2018. “Advances in Antarctic Research for Antimicrobial Discovery: A Comprehensive Narrative Review of Bacteria From Antarctic Environments as Potential Sources of Novel Antibiotic Compounds Against Human Pathogens and Microorganisms of Industrial Importance.” Antibiotics 7: 90. 30347637 10.3390/antibiotics7040090 PMC6316688

[emi470249-bib-0078] Nurk, S. , D. Meleshko , A. Korobeynikov , and P. A. Pevzner . 2017. “metaSPAdes: A New Versatile Metagenomic Assembler.” Genome Research 27: 824–834. 28298430 10.1101/gr.213959.116 PMC5411777

[emi470249-bib-0079] O'Leary, N. A. , M. W. Wright , J. R. Brister , et al. 2016. “Reference Sequence (RefSeq) Database at NCBI: Current Status, Taxonomic Expansion, and Functional Annotation.” Nucleic Acids Research 44: D733–D745. 26553804 10.1093/nar/gkv1189 PMC4702849

[emi470249-bib-0080] Ondon, B. S. , S. Li , Q. Zhou , and F. Li . 2021. “Sources of Antibiotic Resistant Bacteria (ARB) and Antibiotic Resistance Genes (ARGs) in the Soil: A Review of the Spreading Mechanism and Human Health Risks.” In Reviews of Environmental Contamination and Toxicology, edited by P. de Voogt , vol. 256, 121–153. Springer International Publishing. 33948742 10.1007/398_2020_60

[emi470249-bib-0081] Ortiz, M. , P. M. Leung , G. Shelley , et al. 2021. “Multiple Energy Sources and Metabolic Strategies Sustain Microbial Diversity in Antarctic Desert Soils.” Proceedings of the National Academy of Sciences of the United States of America 118: e2025322118. 34732568 10.1073/pnas.2025322118 PMC8609440

[emi470249-bib-0082] Pantůček, R. , I. Sedláček , A. Indráková , et al. 2018. “ *Staphylococcus edaphicus* sp. nov., Isolated in Antarctica, Harbors the mecC Gene and Genomic Islands With a Suspected Role in Adaptation to Extreme Environments.” Applied and Environmental Microbiology 84: e01746‐17. 29079617 10.1128/AEM.01746-17 PMC5752872

[emi470249-bib-0083] Parks, D. H. , M. Imelfort , C. T. Skennerton , P. Hugenholtz , and G. W. Tyson . 2015. “CheckM: Assessing the Quality of Microbial Genomes Recovered From Isolates, Single Cells, and Metagenomes.” Genome Research 25: 1043–1055. 25977477 10.1101/gr.186072.114 PMC4484387

[emi470249-bib-0084] Quinn, G. A. , and P. J. Dyson . 2024. “Going to Extremes: Progress in Exploring New Environments for Novel Antibiotics.” Npj Antimicrobial Resistance 2: 8. 10.1038/s44259-024-00025-8 PMC11721673 39843508

[emi470249-bib-0085] R Core Team . 2024. R: A Language and Environment for Statistical Computing. R Foundation for Statistical Computing.

[emi470249-bib-0086] Rawat, M. , M. Chauhan , and A. Pandey . 2024. “Extremophiles and Their Expanding Biotechnological Applications.” Archives of Microbiology 206: 247. 38713374 10.1007/s00203-024-03981-x

[emi470249-bib-0087] Richter, I. , C. W. Herbold , C. K. Lee , I. R. McDonald , J. E. Barrett , and S. C. Cary . 2014. “Influence of Soil Properties on Archaeal Diversity and Distribution in the McMurdo Dry Valleys, Antarctica.” FEMS Microbiology Ecology 89: 347–359. 24646164 10.1111/1574-6941.12322

[emi470249-bib-0088] Rizzo, C. , M. Papale , and A. Lo Guidice . 2021. “New Trends in Antarctic Bioprospecting: The Case of Cold‐Adapted Bacteria.” In Extreme Environments: Unique Ecosystems – Amazing Microbes, edited by A. Pandey and A. Sharma . CRC Press, Taylor and Francis Group.

[emi470249-bib-0089] Salzberg, S. L. 2019. “Next‐Generation Genome Annotation: We Still Struggle to Get It Right.” Genome Biology 20: 92. 31097009 10.1186/s13059-019-1715-2 PMC6521345

[emi470249-bib-0090] Scales, N. C. , K. T. Huynh , C. Weihe , and J. B. H. Martiny . 2023. “Desiccation Induces Varied Responses Within a Soil Bacterial Genus.” Environmental Microbiology 25: 3075–3086. 37664956 10.1111/1462-2920.16494

[emi470249-bib-0091] Schloss, P. D. , R. A. Girard , T. Martin , J. Edwards , and J. C. Thrash . 2016. “Status of the Archaeal and Bacterial Census: An Update.” MBio 7: e00201‐16. 10.1128/mbio.00201-16. 27190214 PMC4895100

[emi470249-bib-0092] Shaw, E. A. , B. Adams , J. Barrett , W. Lyons , R. Virginia , and D. Wall . 2018. “Stable C and N Isotope Ratios Reveal Soil Food Web Structure and Identify the Nematode *Eudorylaimus antarcticus* as an Omnivore–Predator in Taylor Valley, Antarctica.” Polar Biology 41: 1013–1018.

[emi470249-bib-0093] Shen, W. , S. Le , Y. Li , and F. Hu . 2016. “SeqKit: A Cross‐Platform and Ultrafast Toolkit for FASTA/Q File Manipulation.” PLoS One 11: e0163962. 27706213 10.1371/journal.pone.0163962 PMC5051824

[emi470249-bib-0094] Sieber, C. M. K. , A. J. Probst , A. Sharrar , et al. 2018. “Recovery of Genomes From Metagenomes via a Dereplication, Aggregation and Scoring Strategy.” Nature Microbiology 3: 836–843. 10.1038/s41564-018-0171-1 PMC6786971 29807988

[emi470249-bib-0095] Sikorski, J. 2015. “The Prokaryotic Biology of Soil.” Soil Organisms 87: 1–28.

[emi470249-bib-0096] Simmons, B. L. , D. H. Wall , B. J. Adams , E. Ayres , J. E. Barrett , and R. A. Virginia . 2009. “Long‐Term Experimental Warming Reduces Soil Nematode Populations in the McMurdo Dry Valleys, Antarctica.” Soil Biology & Biochemistry 41: 2052–2060.

[emi470249-bib-0097] Smith, T. E. , D. H. Wall , I. D. Hogg , B. J. Adams , U. N. Nielsen , and R. A. Virginia . 2012. “Thawing Permafrost Alters Nematode Populations and Soil Habitat Characteristics in an Antarctic Polar Desert Ecosystem.” Pedobiologia 55: 75–81.

[emi470249-bib-0098] Sokol, E. R. , C. W. Herbold , C. K. Lee , S. C. Cary , and J. E. Barrett . 2013. “Local and Regional Influences Over Soil Microbial Metacommunities in the Transantarctic Mountains.” Ecosphere 4: 136.

[emi470249-bib-0099] Spagnolo, F. , M. Trujillo , and J. J. Dennehy . 2021. “Why Do Antibiotics Exist?” MBio 12: e01966‐21. 34872345 10.1128/mBio.01966-21 PMC8649755

[emi470249-bib-0100] Thompson, A. R. , S. Geisen , and B. J. Adams . 2020. “Shotgun Metagenomics Reveal a Diverse Assemblage of Protists in a Model Antarctic Soil Ecosystem.” Environmental Microbiology 22: 4620–4632. 32803809 10.1111/1462-2920.15198

[emi470249-bib-0101] Thompson, A. R. , A. J. Roth‐Monzón , Z. T. Aanderud , and B. J. Adams . 2021. “Phagotrophic Protists and Their Associates: Evidence for Preferential Grazing in an Abiotically Driven Soil Ecosystem.” Microorganisms 9: 1555. 34442632 10.3390/microorganisms9081555 PMC8398437

[emi470249-bib-0102] Van Horn, D. J. , M. L. Van Horn , J. E. Barrett , et al. 2013. “Factors Controlling Soil Microbial Biomass and Bacterial Diversity and Community Composition in a Cold Desert Ecosystem: Role of Geographic Scale.” PLoS One 8: e66103. 23824063 10.1371/journal.pone.0066103 PMC3688848

[emi470249-bib-0103] Varliero, G. , P. H. Lebre , B. Adams , et al. 2024. “Biogeographic Survey of Soil Bacterial Communities Across Antarctica.” Microbiome 12: 9. 38212738 10.1186/s40168-023-01719-3 PMC10785390

[emi470249-bib-0104] Virginia, R. A. , and D. H. Wall . 1999. “How Soils Structure Communities in the Antarctic Dry Valleys.” BioScience 49: 973–983.

[emi470249-bib-0105] Vos, M. , A. B. Wolf , S. J. Jennings , and G. A. Kowalchuk . 2013. “Micro‐Scale Determinants of Bacterial Diversity in Soil.” FEMS Microbiology Reviews 37: 936–954. 23550883 10.1111/1574-6976.12023

[emi470249-bib-0106] Vosloo, S. , L. Huo , C. L. Anderson , Z. Dai , M. Sevillano , and A. Pinto . 2021. “Evaluating de Novo Assembly and Binning Strategies for Time Series Drinking Water Metagenomes.” Microbiology Spectrum 9: e01434‐21. 34730411 10.1128/Spectrum.01434-21 PMC8567270

[emi470249-bib-0107] Wang, H. , R. Bier , L. Zgleszewski , et al. 2020. “Distinct Distribution of Archaea From Soil to Freshwater to Estuary: Implications of Archaeal Composition and Function in Different Environments.” Frontiers in Microbiology 11: 11–2020. 10.3389/fmicb.2020.576661 PMC7642518 33193193

[emi470249-bib-0108] Waschulin, V. , C. Borsetto , R. James , et al. 2022. “Biosynthetic Potential of Uncultured Antarctic Soil Bacteria Revealed Through Long‐Read Metagenomic Sequencing.” ISME Journal 16: 101–111. 34253854 10.1038/s41396-021-01052-3 PMC8692599

[emi470249-bib-0109] Wei, S. T. S. , C. M. Higgins , E. M. Adriaenssens , D. A. Cowan , and S. B. Pointing . 2015. “Genetic Signatures Indicate Widespread Antibiotic Resistance and Phage Infection in Microbial Communities of the McMurdo Dry Valleys, East Antarctica.” Polar Biology 38: 919–925.

[emi470249-bib-0110] Wickham, H. 2016. ggplot2: Elegant Graphics for Data Analysis. Springer‐Verlag.

[emi470249-bib-0111] Wu, D. , R. Seshadri , N. C. Kyrpides , and N. N. Ivanova . 2025. “A Metagenomic Perspective on the Microbial Prokaryotic Genome Census.” Science Advances 11: eadq2166. 39823337 10.1126/sciadv.adq2166 PMC11740963

[emi470249-bib-0112] Wu, Y.‐W. , B. A. Simmons , and S. W. Singer . 2016. “MaxBin 2.0: An Automated Binning Algorithm to Recover Genomes From Multiple Metagenomic Datasets.” Bioinformatics 32: 605–607. 26515820 10.1093/bioinformatics/btv638

[emi470249-bib-0113] Xia, L. C. , J. A. Cram , T. Chen , J. A. Fuhrman , and F. Sun . 2011. “Accurate Genome Relative Abundance Estimation Based on Shotgun Metagenomic Reads.” PLoS One 6: e27992. 22162995 10.1371/journal.pone.0027992 PMC3232206

[emi470249-bib-0114] Xue, X. , A. R. Thompson , and B. J. Adams . 2024. “An Antarctic Worm and Its Soil Ecosystem: A Review of an Emerging Research Program in Ecological Genomics.” Applied Soil Ecology 193: 105110.

[emi470249-bib-0115] Zablocki, O. , L. van Zyl , E. M. Adriaenssens , et al. 2014. “High‐Level Diversity of Tailed Phages, Eukaryote‐Associated Viruses, and Virophage‐Like Elements in the Metaviromes of Antarctic Soils.” Applied and Environmental Microbiology 80: 6888–6897. 25172856 10.1128/AEM.01525-14 PMC4249006

[emi470249-bib-0116] Zhang, Y. , and C. A. Gross . 2021. “Cold Shock Response in Bacteria.” Annual Review of Genetics 55: 377–400. 10.1146/annurev-genet-071819-031654 34530639

